# 5,12‐TIPS‐Substitution of Tetracene: Effects on Redox Potentials and Cyclic Voltammetric Behavior in CH_2_Cl_2_ and THF^1^

**DOI:** 10.1002/chem.202503591

**Published:** 2026-05-09

**Authors:** Holger F. Bettinger, Simon Schundelmeier, Bernd Speiser

**Affiliations:** ^1^ Institut für Organische Chemie Universität Tübingen Tübingen Germany

**Keywords:** arenes, cyclic voltammetry, density functional calculations, molecular electrochemistry, radical ions, redox processes, solvent effects, substituent effects

## Abstract

Tetracene and 5,12‐bis(tri(isopropyl)silylethynyl)tetracene (TIPS‐tetracene) are widely used in optoelectronic applications. Their electrochemical behavior is compared in the present work by cyclic voltammetry and ESR‐spectroelectrochemistry. Their electrode reaction mechanisms and relevant physico‐chemical parameters are derived for ${\mathrm{CH}}_{2}$ ${\mathrm{Cl}}_{2}$‐ and THF‐based electrolytes from experimental data. DFT calculations provide energies of the acenes and their radical ions in gas phase and solution environments. The differences between the behavior of the compounds and between the solvent conditions are discussed. The effect of the two TIPS substituents on the formal potentials of the (first) one‐electron oxidation and reduction steps in the two solvents and on the kinetic stabilization of the radical ions is evaluated. As a global redox characteristic, an “electrochemical gap” is discussed for the tetracenes under the solvent conditions.

## Graphical abstract

Tetracene and bis(tri(isopropyl)silylethynyl)tetracene (TIPS‐tetracene) exhibit characteristic similarities and differences in their electrochemical redox behavior depending on the base solvent of the electrolyte. Analysis of cyclic voltammograms allows the quantification of substituent and solvent effects and the calculation of an “electrochemical gap.”

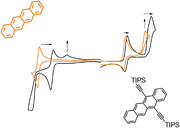

## 1 Introduction

Materials based on organic molecules are being developed for several decades [1, 2, 3], and have found (or let us envisage [4]) applications in technological fields, such as light emission [5] and photovoltaics [6] or spintronics [7]. Owing to solubility and processability (supporting, for example, ink‐jet techniques on flexible substrates) as well as efficient synthetic approaches, organic materials present an alternative to their inorganic counterparts [8].

**FIGURE 1:**
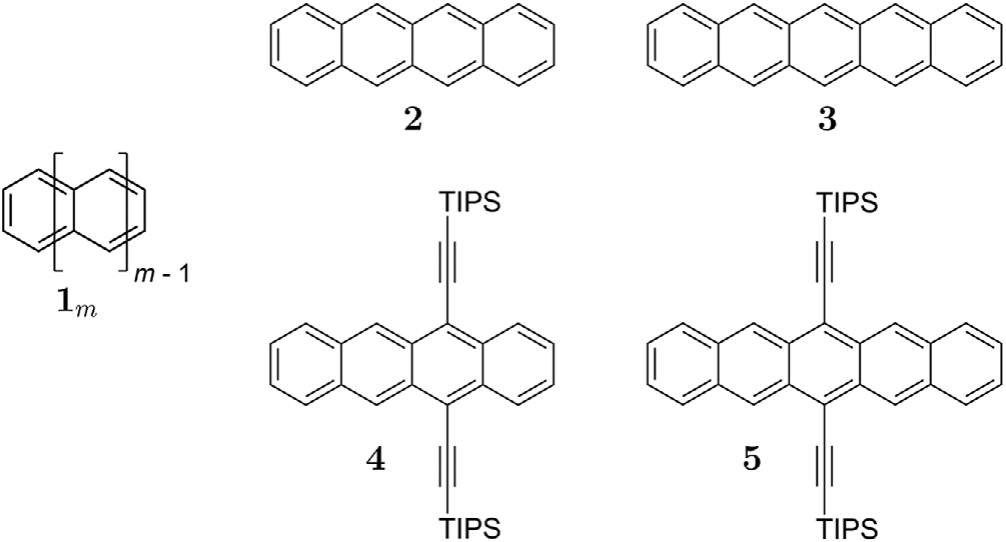
Chemical structures of a generalized acene and important acenes investigated in this work.

A certain class of organic molecules that has become central to optoelectronics [8], are linearly annulated aromatic ring systems, the acenes **1** 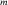 (see, Figure 1) [9, 10].

Acene applications [8] exploit the small energetic gap [10] between the highest occupied (HOMO) and the lowest unoccupied (LUMO) molecular orbitals, as well as the high charge carrier mobility [11, 12]. Advantageous supramolecular structures in the solid state [8, 13] lead to high electronic conductivity and applications in organic field effect transistors (OFETs), organic light emitting diodes (OLEDs), sensors, photovoltaic devices, and more [3]. The singlet fission phenomenon [14, 15] has been observed for acenes [16, 17], which offers the potential to increase the quantum efficiency of organic solar cells [18, 19] over the Shockley–Queisser limit [14]. Most recently, radical states of acene materials were discussed in the context of quantum technology [20] and quantum information science [21], adding further opportunities for applications. Acenes are also regarded as low molecular weight fragments of graphene, and indeed “graphenic radicals” and their redox behavior show some similarities to acenes [22].

The desired electronic properties of acenes improve with the number of annulated rings (acene length [8, 23]), m. However, the stability and solubility, important for processing the respective materials, decrease with m. Most applications thus employ tetracene **2** (**1** 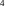) and pentacene **3** (**1** 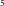) as a compromise.

In an important innovation, Anthony suggested to substitute H‐atoms in the acene core with tri(isopropyl)silylethynyl groups [24]. For 5,12‐bis(tri(isopropyl)silylethynyl)tetracene (TIPS‐tetracene, **4**) [25] and 6,13‐bis(tri(isopropyl)silylethynyl)pentacene (TIPS‐pentacene, **5**) [24], dramatic increases of both stability and solubility were observed. For TIPS‐pentacene, the advantageous “bricklayer” structure [13] provides enhanced π‐interaction between the individual molecules in the solid state and consequently improves electric conductivity as compared to the unsubstituted parent molecule. TIPS‐pentacene has become a de facto standard for many investigations of acenes. Ethynyl substitution is also employed for linking acene subunits [26, 27, 28]. It is interesting to note that such ethynyl bridges extend the electronic acene structure [28]. This demonstrates that the ethynyl substituent might exert considerable effects on electronic properties. The exact nature of these effects is, however, complex [29].

A popular experimental technique to investigate relevant optoelectronic properties is cyclic voltammetry (CV) [30, 31, 32]. It is common to include CV data in acene characterization (see for example refs. [33, 34, 35, 36]). While scanning the electrode potential as a triangular function of time and recording the current, we induce redox reactions of the investigated compound(s) at the electrode and detect redox‐active products. We will use CV in the following in liquid, stationary electrolytes and at a solid disk electrode. We observe the potential and current features of the voltammetric signals, the “peaks,” and derive information about the redox behavior and electrode mechanisms, such as formal redox potentials, electron transfer rate constants, and transport properties.

Of particular interest in the present context is the determination of redox potentials that characterize the thermodynamics of individual electron transfer steps

(1) $$\text{Red}-e^{-}\;\;\;⇌\;\;\;\text{Ox}$$

(Red and Ox are the reduced and oxidized forms of a redox couple, respectively) from experimental data. Certain prerequisites are essential to arrive at valid results, such as the electron transfer being reversible. In the field of molecular electrochemistry, reversibility is defined by requiring the redox couple Red/Ox to be in Nernst equilibrium at the electrode surface (see entry “c” under term “reversible redox reaction” in ref. [37]; “electrochemical reversibility”). This condition applies if the electron transfer kinetics is sufficiently fast and transport processes to and from the electrode are rate‐determining. Transport in CV is restricted to diffusion since the supporting electrolyte concentration is conventionally high enough to eliminate migration and the electrolyte is at rest to make convection negligible. Therefore, this situation is also often termed “diffusion controlled.” It is also advantageous if any chemical transformations of the redox states exert only negligible effects by proceeding only slowly at the time scale of the experiment (“chemical reversibility”). The time scale of CV is controlled by the potential scan rate v. Verification of the pre‐conditions requires detailed studies of the underlying electrochemical and chemical steps, judicious selection of the experimental parameters of the CV experiment, most prominently v and the substrate concentration c, as well as careful data analysis. Unfortunately, many papers in the literature lack such considerations and use only approximate or less strictly defined measures of redox potentials, such as, for example, the “onset potential” of a voltammetric wave or peak potentials, both of which, however, may not only be controlled by thermodynamics, but also have kinetic and other distorting components to a varying extent. Moreover, in many cases, only a single combination of scan rate and concentration is used, thus ignoring the most important property of CV that helps to elucidate kinetics and mechanisms by changing the experimental time scale and c.

In the present paper, we will compare the cyclic voltammetric features of TIPS‐tetracene **4** and tetracene **2** as the main goal. This will provide an electrochemical benchmark for the substituent effect of the tri(isopropyl)silylethynyl substituent on the parent acene. Experiments will be performed in electrolytes, which are based on the nonaqueous solvents dichloromethane (${\mathrm{CH}}_{2}$ ${\mathrm{Cl}}_{2}$) and tetrahydrofuran (THF) with the tetra‐*n*‐butylammonium hexafluorophosphate (${\mathrm{NBu}}_{4}$ ${\mathrm{PF}}_{6}$) supporting electrolyte. Dichloromethane is a commonly used nonaqueous solvent for electroanalytical studies, also popular in the acene field [25, 35, 38, 39, 40, 41]. THF is another polar nonaqueous solvent employed in electrochemistry, which exhibits a particularly wide electrochemical window in the negative potential range. However, in THF, we often find increased IR drop [42]. This artifact results from diminished conductivity of the solution, probably due to association of the electrolyte ions in the nonaqueous solvents, and hampers the analysis of the data. Association of electrolyte ions has been observed in both ${\mathrm{CH}}_{2}$ ${\mathrm{Cl}}_{2}$ and THF [43]. However, the association tendency is much larger in THF. A higher supporting electrolyte concentration in THF as compared to the common 0.1 M ${\mathrm{NBu}}_{4}$ ${\mathrm{PF}}_{6}$ may compensate for the decreased dissociation of the salt in THF and keep the conductance of the solution sufficiently high to avoid excessive solution resistance R and consequently IR drop. An increase of the concentration to 0.2 M has been found earlier in our group to adequately mitigate the problem if instrumental IR compensation is used as well (see Experimental Part). For reasons of comparison, also experiments in 0.2 M ${\mathrm{NBu}}_{4}$ ${\mathrm{PF}}_{6}$/${\mathrm{CH}}_{2}$ ${\mathrm{Cl}}_{2}$ will be reported in the following. Thus, the effect of the supporting electrolyte concentration in dichloromethane can also be estimated. Additional comparisons to TIPS‐pentacene electrochemistry [36, 44] will integrate the results with those of other acenes. The formal potentials that are determined under reversible conditions do also allow the estimation of an “electrochemical gap” between the primary oxidation and reduction reactions of the tetracenes (note the cautious remarks given in ref. [45] for the interpretation of such a property and its relation to the HOMO–LUMO gap). An additional approach to substituent and solvent effects is finally provided by DFT calculations of ionization energies and electron affinities of the acenes and their radical ions.

Comparing the electrochemical results for the substituted and the unsubstituted tetracene will further allow to evaluate the impact of the TIPS substituent, often seen as a protecting group [8, 46], not only on redox potentials and solvent effects but also on reactivity of electron transfer products. We have already demonstrated that TIPS is not innocent with respect to reaction of the primary oxidation product of **5** on longer (electrolysis) time scales [44]. Analogous reactivity has been found just recently for an acid‐mediated reaction as well [47]. In the present paper, we will deal with shorter (voltammetric) time scales.

## 2 Results and Discussion

### 2.1 Overview of Tetracene and TIPS‐Tetracene Redox Processes

**FIGURE 2:**
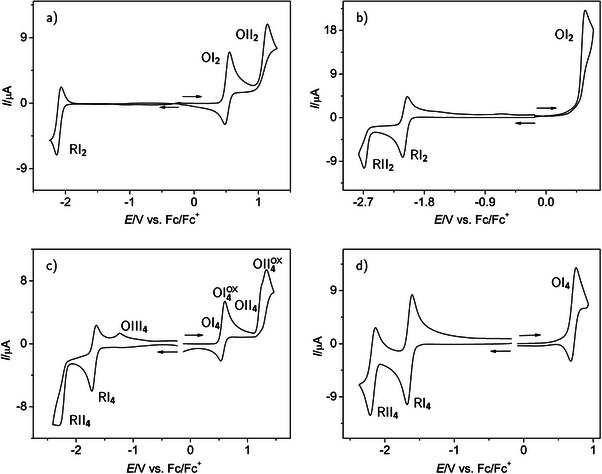
Overview cyclic voltammograms of tetracene **2** (a,b) and 5,12‐bis(tri(isopropyl)silylethynyl)tetracene (TIPS‐tetracene) **4** (c,d) in the complete electrochemical window of the 0.1 M ${\mathrm{NBu}}_{4}$ ${\mathrm{PF}}_{6}$/${\mathrm{CH}}_{2}$ ${\mathrm{Cl}}_{2}$ (a,c) and 0.2 M ${\mathrm{NBu}}_{4}$ ${\mathrm{PF}}_{6}$/THF (b,d) electrolytes, v=0.2 V $s^{-1}$; (a) c=0.22 mM, (b) c=0.27 mM, (c) c=0.25 mM, (d) c=0.58 mM; two cycles starting at the rest potential are combined in each graph; arrows indicate the direction of the forward scans of the individual voltammograms.

Cyclic voltammograms of the unsubstituted tetracene **2** (Figure 2a,b) and 5,12‐bis(tri(isopropyl)silylethynyl)tetracene **4** (Figure 2c,d) in the entire accessible potential range (“potential window”) of the respective electrolyte provide an overview and supply initial qualitative^2^ information about all observable redox processes. The curves presented in Figure 2 are composed of two cycles each. The cycles scan the potential from a value close to the rest potential of the solution with the tetracene or TIPS‐tetracene analyte (i.e., the potential where only a negligible current flows and conversion of the starting acene at the electrode is absent) to either the positive or negative limit of the potential window, where the background current strongly increases even without analyte. Since background corrected voltammograms are displayed, this artifact is mostly eliminated in the curves (for an example of the effect of background correction, see Figure S1). We compare the overview voltammograms in electrolytes based on ${\mathrm{CH}}_{2}$ ${\mathrm{Cl}}_{2}$ (with a 0.1 M concentration of the supporting electrolyte ${\mathrm{NBu}}_{4}$ ${\mathrm{PF}}_{6}$) and on THF (with a 0.2 M concentration of the same supporting electrolyte).

The rest potential of the Pt disk electrode in the ${\mathrm{CH}}_{2}$ ${\mathrm{Cl}}_{2}$ electrolyte is −0.21 V versus the Fc/${\mathrm{Fc}}^{+}$ (Fc = ferrocene) redox couple, while it is −0.25 V in the THF electrolyte. All potentials in this paper are reported with respect to the ferrocene reference value in the respective electrolyte. Separate (external) determination of the ferrocene potential avoids possible interactions of the reference compound with the redox active acene or its electron transfer products in the solution.

**FIGURE 3:**
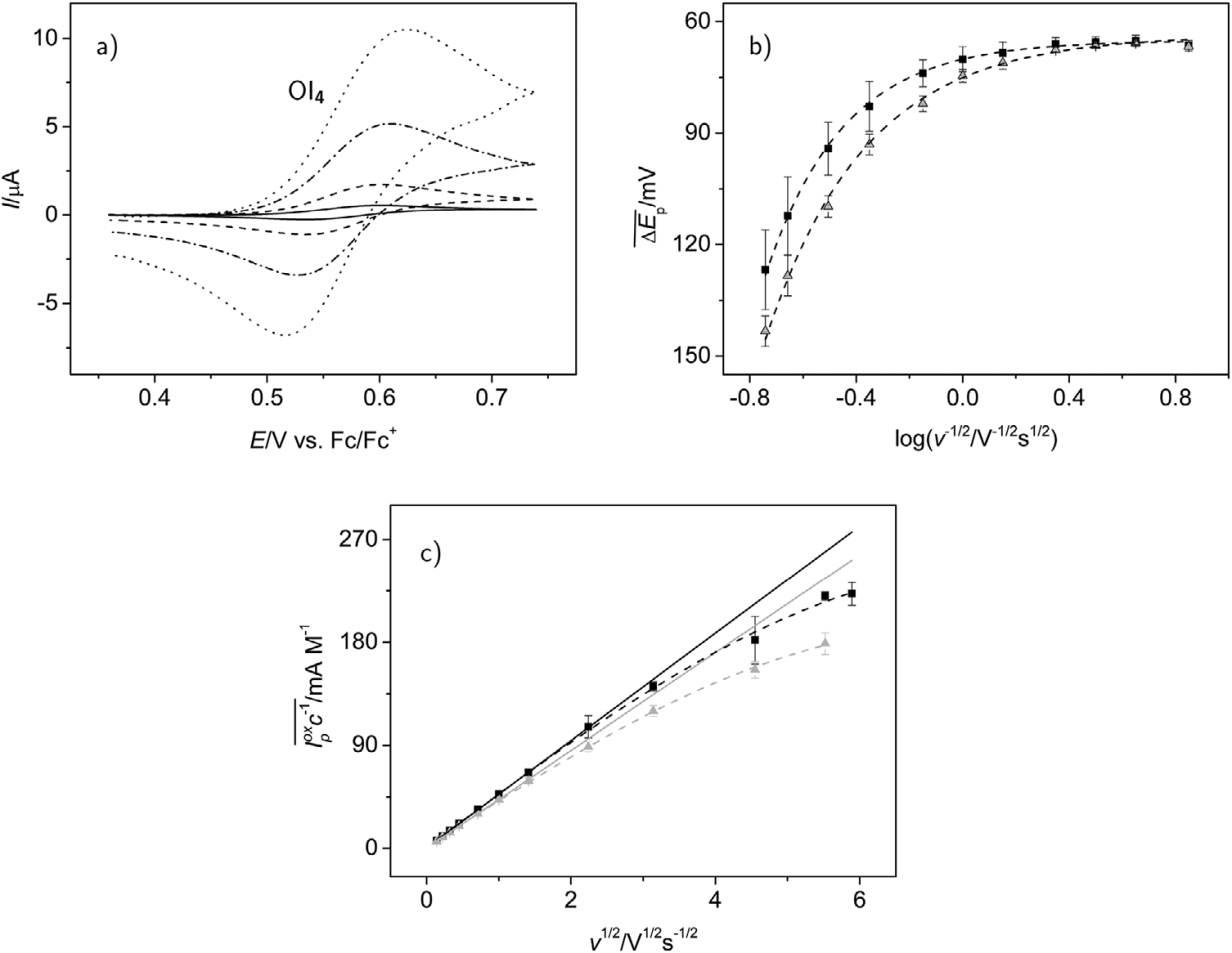
Cyclic voltammetry of TIPS‐tetracene **4** in the potential range of the first oxidation in ${\mathrm{NBu}}_{4}$ ${\mathrm{PF}}_{6}$/${\mathrm{CH}}_{2}$ ${\mathrm{Cl}}_{2}$ electrolytes; (a) selected cyclic voltammograms (c=0.085 mM, v=0.02, 0.2, 2.0, and 10 V $s^{-1}$ with increasing intensity of the signal), supporting electrolyte concentration: 0.1 M; (b) variation of peak potential difference $\Delta E_{p}$(${\mathrm{OI}}_{4}$) with scan rate v, black squares: supporting electrolyte concentration 0.1 M, mean values of three independent measurements with 17 concentrations, 0.043 ≤c/mM≤0.24, gray triangles: electrolyte concentration 0.2 M, mean values of two independent measurements with 10 concentrations, 0.034 ≤c/mM≤0.21, the plot is arranged in analogy to Fig. 3 in ref. [61], where the logarithmic x axis features a quantity ψ, proportional to $v^{-1/2}$; (c) variation of the concentration normalized oxidation peak currents $I_{p}^{\mathrm{ox}}\left({\mathrm{OI}}_{4}\right)c^{-1}$ with $\sqrt{v}$, black squares and gray triangles as in (b), straight lines extrapolate the linear dependency found for small scan rates; the broken lines in (b) and (c) are only given as guide for the eye and do not carry physical meaning; error bars in (b) and (c): standard deviations.

Both compounds show oxidation as well as reduction processes in both solvents. However, the number of visible processes and their gross characteristics differ. In contrast, there is no such difference between cyclic overview voltammograms in ${\mathrm{CH}}_{2}$ ${\mathrm{Cl}}_{2}$ electrolytes with 0.1 M (Figure 2) and 0.2 M (shown for the example of the tetracene oxidation part of the voltammogram in Figure S4) concentrations of ${\mathrm{NBu}}_{4}$ ${\mathrm{PF}}_{6}$. Note, however, the quantitative differences in voltammetric features and reaction parameters caused by the different supporting electrolyte concentrations as shown in Figures 3b,c and 5b,c as well as Tables 1 and 3. In the following, we denote redox processes with “O” and “R” for initial oxidation or reduction during a voltammetric cycle, and assign roman numbers according to the order of appearance within the cycle. The number of the compound involved is appended as a subscript.

TIPS‐tetracene **4** shows two oxidation processes ${\mathrm{OI}}_{4}$ and ${\mathrm{OII}}_{4}$ during a cycle with initially *positive* scan direction in the ${\mathrm{CH}}_{2}$ ${\mathrm{Cl}}_{2}$ electrolyte (Figure 2c), of which the one at less positive potentials features a reverse peak even at the relatively small scan rate of 0.2 V $s^{-1}$. Thus, the primary product of the electron transfer appears to have a reasonable life time to be detected on the reverse scan. At the given scan rate the second oxidation process is not chemically reversible, that is, at this time scale of the experiment some chemical follow‐up reaction completely removes the product of process ${\mathrm{OII}}_{4}$ from the diffusion layer at the electrode causing the reverse peak to be absent. The respective second oxidation signal ${\mathrm{OII}}_{4}^{\mathrm{ox}}$ (the superscript indicates the direction of electron transfer causing the peak signal) is clearly more intense than the first oxidation signal, even if we consider the correct baseline (extrapolation of the decay of current from peak ${\mathrm{OI}}_{4}^{\mathrm{ox}}$). The shoulder in its low potential flank indicates that it might be composed of two closely spaced peaks.

If we record a cycle with the initial scan going to more *negative* potentials, two reduction processes ${\mathrm{RI}}_{4}$ and ${\mathrm{RII}}_{4}$ (the latter clearly without chemical reversibility) are observed. After scanning the potential through the region of irreversible process ${\mathrm{RII}}_{4}$, we find a follow‐up process ${\mathrm{OIII}}_{4}$, identified by a peak with minor intensity. Similar signals have been observed for TIPS‐pentacene **5** [36] and other oligoaromatic compounds (e.g., anthracene [48], pentacene derivatives (see Figure S9 in the Supporting Information in ref. [49]), or indacene‐tetrathiafulvalenes (see supporting information in ref. [50])). The corresponding oxidizable species is generated while scanning through the second reduction peak of the acene, because it is not observed when we switch the potential scan direction at a potential between the two reduction peaks. This observation is in accordance with the earlier work in the literature. There, the product of mono‐protonation of the highly basic dianion was assumed to be responsible for the oxidation peak [48, 50]. We regard the involvement of water as a proton source and protonation of the dianion as highly unlikely, because experiments with TIPS‐pentacene in ${\mathrm{CH}}_{2}$ ${\mathrm{Cl}}_{2}$ in the presence of deliberately added water did show this peak with reduced intensity.

In the THF electrolyte, the primary oxidation of **4** (process ${\mathrm{OI}}_{4}$, Figure 2d) is already close to the positive limit of the potential window (approximately +0.8 V), but a reverse peak is still visible. In the negative potential region, two obviously chemically rather reversible processes ${\mathrm{RI}}_{4}$ and ${\mathrm{RII}}_{4}$ appear. Although potentials down to approximately −3.5 V can be reached in the THF electrolyte [36], no further signals were observed at potentials more negative than −2.4 V. A follow‐up product signal as in the ${\mathrm{CH}}_{2}$ ${\mathrm{Cl}}_{2}$ electrolyte is not apparent. The signal of ${\mathrm{RII}}_{4}$ has no longer an irreversible shape as in ${\mathrm{CH}}_{2}$ ${\mathrm{Cl}}_{2}$. This indicates that the reaction of the reduction product species and the appearance of ${\mathrm{OIII}}_{4}$ in Figure 2c must be related to the solvent identity. Although we can not exclude the possibility that the intrinsic stability of **4** 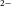 is very different in the two solvents used here, it seems highly probable that some reduction product formed from **4** reacts with ${\mathrm{CH}}_{2}$ ${\mathrm{Cl}}_{2}$ at the rather negative potentials of processes like ${\mathrm{RII}}_{4}$ (close to the negative limit of accessible potentials), but not with THF.

It is interesting to note that the redox processes in THF are shifted positive by up to 100 mV or more as compared to the respective processes in the ${\mathrm{CH}}_{2}$ ${\mathrm{Cl}}_{2}$ electrolyte. As a result, a hypothetical second oxidation in THF might be shifted out of the accessible potential window.

The electrochemical oxidation and/or reduction of **4** has been observed on several occasions in a variety of solvents and with various supporting electrolytes, but most often only a single oxidation and/or reduction process is mentioned [25, 51, 52]. Redox potentials are in some cases reported to only 0.1 V, with a quoted uncertainty of not better than 0.05 V [39]. More detailed analyses of the cyclic voltammograms are usually omitted. However, already the overview voltammograms of **4** in the present work show that the redox behavior of the substituted TIPS‐tetracene is complex and, depending on the solution environment, not only one‐electron products but also additional oxidation states are available by electrochemical electron transfer.

The cyclic voltammograms of unsubstituted tetracene **2** (Figure 2a,b) show similarities but also some characteristic differences compared to those of **4**. In the ${\mathrm{CH}}_{2}$ ${\mathrm{Cl}}_{2}$ electrolyte, we again observe two oxidation processes ${\mathrm{OI}}_{2}$ and ${\mathrm{OII}}_{2}$. At least the signal at more positive potentials indicates some involvement of a chemical follow‐up reaction at 0.5 V $s^{-1}$ (Figure 2a) through a missing (${\mathrm{OII}}_{2}$) reverse peak. In the region of negative potentials, where **2** is reduced, we find only a single, obviously chemically near‐reversible process ${\mathrm{RI}}_{2}$. On the other hand, based on the absence of the respective reverse peak, in the THF electrolyte process ${\mathrm{OI}}_{2}$ is chemically irreversible, as is the second reduction process ${\mathrm{RII}}_{2}$ (Figure 2b). The first reduction process ${\mathrm{RI}}_{2}$ seems to remain close to chemically reversible also in THF. It is obvious from the overview voltammograms of **4** and **2** that the reduction processes are shifted to less negative potentials upon TIPS substitution by several 100 mV, while the oxidation signals are much less affected.

Voltammetric results for the electron transfer reactions of unsubstituted tetracene were previously reported for various electrochemical techniques, solvents, supporting electrolytes, and electrode materials [53, 54, 55, 56, 57]. However, the question of chemical reversibility and the observation of a second oxidation process was not unequivocally answered from the literature. For example, in the oxidation of tetracene in ${\mathrm{CH}}_{2}$ ${\mathrm{Cl}}_{2}$/0.2 M ${\mathrm{NBu}}_{4}$ ${\mathrm{ClO}}_{4}$, the instability of the one‐electron oxidation product of the hydrocarbon was mentioned [56]. Temperatures of −70 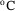 were necessary [57] to observe stabilization of that oxidation state in ${\mathrm{CH}}_{2}$ ${\mathrm{Cl}}_{2}$/0.2 M ${\mathrm{NBu}}_{4}$ ${\mathrm{BF}}_{4}$. However, a second oxidation is not mentioned in either work. On the other hand, two oxidation waves were mentioned for tetracene in unbuffered acetonitrile with 0.1 M ${\mathrm{NaClO}}_{4}$ at a vibrating Pt electrode [55], in ${\mathrm{CH}}_{3}\mathrm{CN}$/0.1 M ${\mathrm{NPr}}_{4}$ ${\mathrm{ClO}}_{4}$ by means of cyclic voltammetry [53] (irreversible second oxidation), and in THF electrolytes at a rotating electrode [54, 58]. As in the case of **4**, detailed analyses of the current‐potential curves with respect to reaction parameters are missing. For any comparison to the substituted TIPS‐tetracene, it will be essential to analyze voltammograms recorded under identical, closely controlled and preferably reversible conditions.

Thus, in the following sections, the individual redox processes will be investigated in more detail and under clearly comparable conditions with respect to thermodynamic, mechanistic, and (electrode‐)kinetic information. The analysis of the first oxidation processes will be emphasized in particular.

### 2.2 TIPS‐Tetracene: First Oxidation Process

#### 2.2.1 Cyclic Voltammetry

By selecting a potential range of +0.359 to +0.739 V in the ${\mathrm{CH}}_{2}$ ${\mathrm{Cl}}_{2}$ electrolyte, we are able to selectively scan the region of the first oxidation process of TIPS‐tetracene, ${\mathrm{OI}}_{4}$. We systematically varied the potential scan rate v between 0.02 and 35 V $s^{-1}$ and the concentration of 4 from 0.043 mM up to 0.24 mM. Voltammograms and plots of important peak features are presented in Figure 3, while characteristic numerical results of the resulting voltammograms are presented in Tables 1 and S1.

**TABLE 1:** Cyclic voltammetric parameters characterizing the first oxidation process of TIPS‐tetracene, ${\mathrm{OI}}_{4}$ (**4**/**4**).

	solvent c(${\mathrm{NBu}}_{4}$ ${\mathrm{PF}}_{6}$)/M	${\mathrm{CH}}_{2}$ ${\mathrm{Cl}}_{2}$		THF
entry		0.1	0.2	0.2
1	$\Delta E_{p}$(${\mathrm{OI}}_{4}$)/V	0.067±0.003 ^a^	0.070±0.003 ^a^	0.078±0.003 ^b^
2	$\Delta E_{p}$(${\mathrm{OI}}_{4}$)/V ^c^	0.112±0.010	0.128±0.005	—
3	$E^{0}$(${\mathrm{OI}}_{4}$)/V ^d^	0.572±0.003 ^e^	0.574±0.001 ^e^	0.715±0.004 ^b^
4	$I_{p}^{\mathrm{red}}\left({\text{OI}}_{4}\right)/I_{p}^{\mathrm{ox}}\left({\text{OI}}_{4}\right)$ ^f^, ^g^	1.00±0.02	0.98±0.02	—
5	$I_{p}^{\mathrm{red}}\left({\text{OI}}_{4}\right)/I_{p}^{\mathrm{ox}}\left({\text{OI}}_{4}\right)$ ^f^, ^h^	0.92±0.03	0.93±0.01	—
6	$I_{p}^{\mathrm{ox}}\left({\text{OI}}_{4}\right)c^{-1}\cdot v^{-1/2}/$	1.50±0.04 ^g^	1.40±0.02 ^i^	—
	mA $s^{1/2}$ $M^{-1}$ ${\mathrm{mV}}^{-1/2}$			
7	$D{\left(4\right)}_{\mathrm{CV}}\cdot 10^{6}$/${\mathrm{cm}}^{2}$ $s^{-1}$ ^i^, ^j^	7.9±0.4	7.0±0.2	—
8	$D{\left(4\right)}_{\mathrm{NMR}}\cdot 10^{6}$/${\mathrm{cm}}^{2}$ $s^{-1}$ ^k^	7.9	7.3	5.6
9	$k_{s}$(${\mathrm{OI}}_{4}$)/${\mathrm{cm}}^{2}$ $s^{-1}$ ^l^	0.06±0.02	0.04±0.003	—

^a^ for small scan rates, 0.05≤v/V $s^{-1}$ ≤1.0, with close to fully reversible shapes of voltammograms, mean values over all concentrations and individual experiments.

^b^ at v=0.2 V $s^{-1}$.

^c^ at v=20 V $s^{-1}$.

^d^ determined as the mid‐point potential.

^e^ for 0.05≤v/V $s^{-1}$ ≤5.0.

^f^ calculated according to Nicholson's equation [59].

^g^ 0.1≤v/V $s^{-1}\leq 5.0$.

^h^ at v=0.05 V $s^{-1}$.

^i^ for 0.05≤v/V $s^{-1}$ ≤1.0.

^j^ from oxidation peak currents according to Randles‐Ševčík equation in the form given by Nicholson and Shain [60].

^k^ from PGSE‐NMR experiments (for details, see Experimental Section).

^l^ from peak potential differences at high scan rates based on ref. [61], v≥5.0 V $s^{-1}$.

At all scan rates, both the oxidation and the reduction peak of process ${\mathrm{OI}}_{4}$ are clearly visible (see Figure 3a for a particular concentration), which allows a quantitative analysis, that is, the determination of parameter values.^2^ At small v, the peak potential differences $\Delta E_{p}\left({\mathrm{OI}}_{4}\right)=E_{p}^{\mathrm{ox}}\left({\mathrm{OI}}_{4}\right)-E_{p}^{\mathrm{red}}\left({\mathrm{OI}}_{4}\right)$ (see Table S1 for a selected experiment with four acene concentrations and c(${\mathrm{NBu}}_{4}$ ${\mathrm{PF}}_{6}$) =0.1 M) remain close to the limiting value of approximately 0.058 V for an electrochemically reversible (diffusion controlled) one‐electron transfer [60]. The values of $\Delta E_{p}$(${\mathrm{OI}}_{4}$) increase with the scan rate (Table S1 and Figure 3b). However, at scan rates up to at least 2.0 V $s^{-1}$, they are essentially independent of the concentration c(**4**). Only at higher scan rates, a mild increase of $\Delta E_{p}$(${\mathrm{OI}}_{4}$) with c(4) becomes apparent. This shows the almost complete compensation of IR drop in the experiment. Thus, the increase of $\Delta E_{p}$(${\mathrm{OI}}_{4}$) with the scan rate indicates the transition from a reversible to a quasi‐reversible process (mixed diffusion and electron transfer control) starting at the time scale given by v≈2.0 V $s^{-1}$. The first two entries in Table 1 displaying $\Delta E_{p}$(${\mathrm{OI}}_{4}$) for the close to reversible situation and, as an example, for the quasi‐reversible case at 20 V $s^{-1}$ support this observation. The peak potential differences in the ${\mathrm{CH}}_{2}$ ${\mathrm{Cl}}_{2}$ electrolytes tend to increase with the concentration of the supporting electrolyte, particularly at the higher scan rates (Figure 3b and Table 1). This effect will be explained further below.

Under reversible or near reversible conditions, formal potential $E^{0}$(${\mathrm{OI}}_{4}$) can be estimated from the mid‐point potential (i.e., the mean value of the oxidation and reduction peak potentials) [30, 62]. Values of this important thermodynamic characteristic of the electron transfer are given as third entry in Table 1 for all electrolytes used. The effect of the supporting electrolyte concentration in ${\mathrm{CH}}_{2}$ ${\mathrm{Cl}}_{2}$ on the formal potential is negligible. Values of ≈+0.6 V versus the Fc/${\mathrm{Fc}}^{+}$ reference for **4** have been reported in the literature for 0.1 M ${\mathrm{NBu}}_{4}$ ${\mathrm{PF}}_{6}$/${\mathrm{CH}}_{2}$ ${\mathrm{Cl}}_{2}$ electrolytes [39, 52] as well as benzonitrile [51] (with unspecified supporting electrolyte).

In the present work, under otherwise comparable conditions, a considerable shift of $E^{0}$(${\mathrm{OI}}_{4}$) by ≈0.14 V to a more positive value is observed if we go from a ${\mathrm{CH}}_{2}$ ${\mathrm{Cl}}_{2}$ to the THF electrolyte. The oxidation of **4** in the THF electrolyte is already rather close to the edge of the accessible potential window. Although the effects of the strongly increasing background current can be eliminated to a large extent by background subtraction (see Experimental Section), the resulting curves were deemed insufficient for more quantitative analysis and some entries in Table 1 are consequently missing. Also, scan rate dependent experiments in the THF electrolyte proved experimentally unsatisfactory owing to electrode fouling, and only v=0.2 V $s^{-1}$ was used.

In ${\mathrm{CH}}_{2}$ ${\mathrm{Cl}}_{2}$ and for scan rates down to 0.1 V $s^{-1}$, the peak current ratio (calculated according to the empirical Nicholson equation [59]; entry 4 in Table 1) remains close to unity, which excludes a chemical follow‐up reaction of the primary oxidation product. Only at v=0.05 V $s^{-1}$ (entry 5) a slight decrease of the ratio is observed. At this time scale, either a slow chemical reaction begins to become significant or edge effects at the finite diameter electrode disk begin to play a role [63].

Concentration normalized peak currents of the oxidation peak are proportional to the square root of the scan rate as predicted for a diffusion‐controlled process by the Randles–Ševčík equation in the formulation by Nicholson and Shain [60] at small to medium scan rates up to 5.0 (0.1 M) or 1.0 V $s^{-1}$ (0.2 M) depending on the supporting electrolyte concentration (Figure 3c). At higher scan rates, there is a deviation from the linear behavior resulting in smaller values of $I_{p}^{\mathrm{ox}}\left({\text{OI}}_{4}\right)c^{-1}$. This is consistent with the hypothesis that the electron transfer becomes quasi‐reversible at faster time scales. If we further normalize the peak currents by the square root of v, the resulting current function [60] is constant for the medium scan rate range (Table 1, entry 6). Adsorption effects, for which the current would increase more strongly than linear with $v^{1/2}$, are absent.

From the peak current values in the diffusion‐controlled time scale range, the diffusion coefficients D(**4**)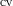 of TIPS‐tetracene in the respective electrolytes are available (Table 1, entry 7) as a parameter defining the transport of **4** in solution. We again use Nicholson and Shain's equation [60], assuming a one‐electron process as derived above from $\Delta E_{p}$. There is a significant decrease of D(4) when increasing the supporting electrolyte concentration in ${\mathrm{CH}}_{2}$ ${\mathrm{Cl}}_{2}$. This is probably due to an increased viscosity of the electrolyte with higher salt concentration. The decrease of D may be the reason for the increase of $\Delta E_{p}$ with c(${\mathrm{NBu}}_{4}$ ${\mathrm{PF}}_{6}$) as apparent from Figure 3b (shift from black to gray curve) and entry 2 in Table 1: slower diffusion results in a more noticeable control by electron transfer kinetics already at slower time scales (small v). We did not determine the diffusion coefficient in THF by CV, again because the signal of process ${\mathrm{OI}}_{4}$ is too close to the positive limit of the accessible potential window. Diffusion coefficients in the ${\mathrm{CH}}_{2}$ ${\mathrm{Cl}}_{2}$ electrolytes were also determined with the pulse gradient spin echo (PGSE‐)NMR technique (Table 1, entry 8) showing good agreement with the electrochemically determined values. In addition, with this methodology, the diffusion coefficient in the THF electrolyte was accessible. Its value is much smaller than that in the ${\mathrm{CH}}_{2}$ ${\mathrm{Cl}}_{2}$ electrolyes, in accordance with the higher viscosity of THF (η=0.456 mPa vs. η=0.413 mPa for ${\mathrm{CH}}_{2}$ ${\mathrm{Cl}}_{2}$ [64]; both values at 25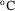).

While the cyclic voltammetric data at slower time scales (small v), where diffusion‐control prevails, were used for the determination of D, at fast time scales (high scan rates), where quasi‐reversibility is observed, information about the electron transfer kinetics becomes available (see Experimental Section) from the peak potential differences [61]. For such an analysis, the value of D in the respective electrolyte must be known. The results (Table 1, entry 9) for the apparent electron transfer rate constant $k_{s}$ tend to decrease with increasing supporting electrolyte concentration. However, the effect is in the order of the standard deviation of the data and thus not regarded as significant.The electron transfer rate constant was not determined from the THF data for the reasons mentioned already above.

In summary, process ${\mathrm{OI}}_{4}$ is a one‐electron transfer, which is electrochemically reversible at small and quasi‐reversible at large scan rates, and should lead to a radical cation **4** 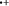. Chemical reactions of this species are negligible at time scales defined by scan rates as low as v=0.05 V $s^{-1}$.

#### 2.2.2 ESR Spectroelectrochemistry of First TIPS‐Tetracene Oxidation Product

**FIGURE 4:**
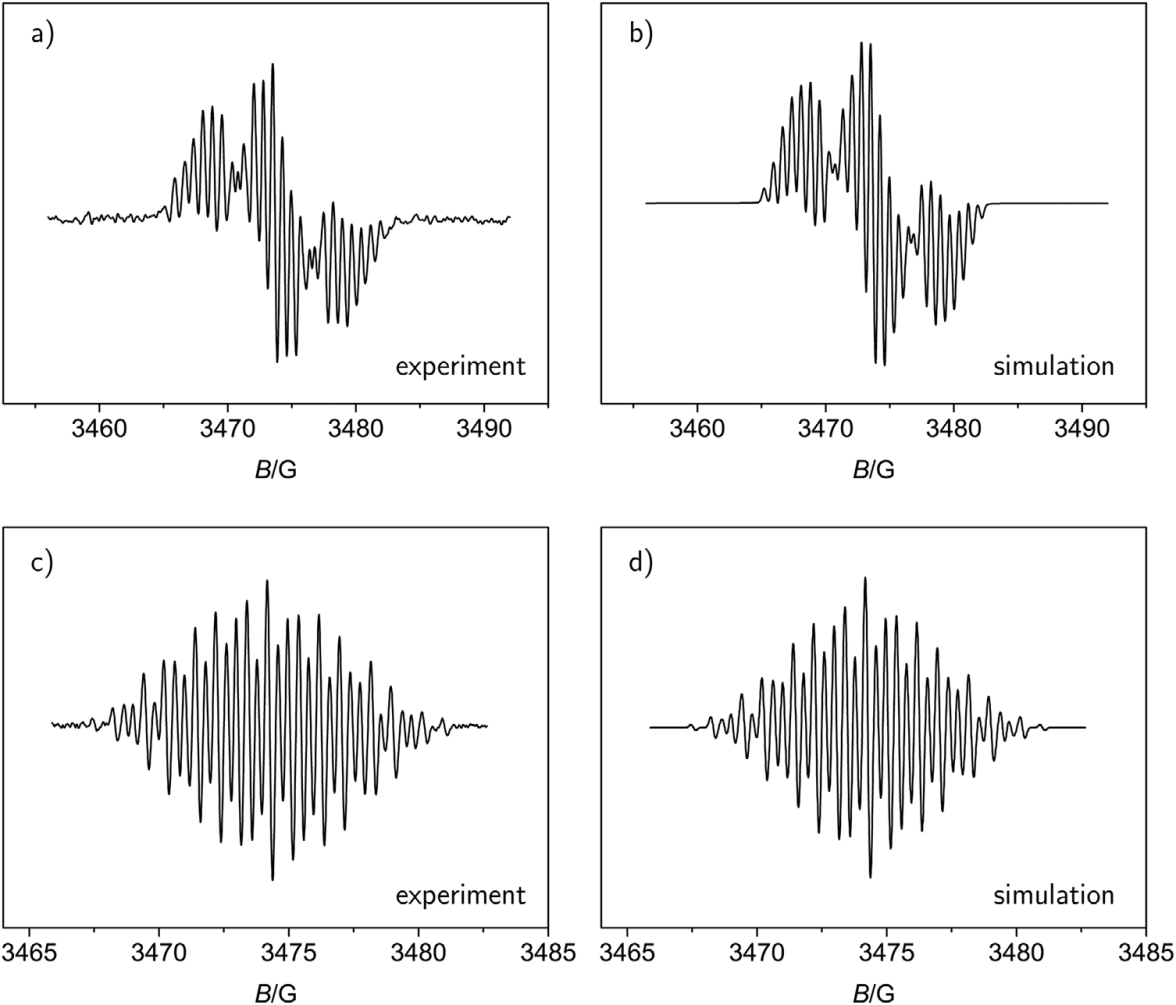
ESR spectra of TIPS‐tetracene radical ions; (a) and (b): **4** 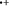, (c) and (d): **4** 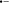; (a) and (c): experimental in 0.1 M ${\mathrm{NBu}}_{4}$ ${\mathrm{PF}}_{6}$/${\mathrm{CH}}_{2}$ ${\mathrm{Cl}}_{2}$; (b) and (d): simulation with parameters as given in Table 2; (a): after electrolysis at a terminal voltage U=2.2 V for 4 min; (c) after electrolysis at U=2.9 V for t=14 min.

The previous results can be confirmed by in situ X‐band ESR spectroscopic detection and characterization of the oxidation product in a flat cell with two electrodes. The terminal voltage U between working and counter electrode was increased until an ESR signal appeared. The shape of the experimental spectrum could be reproduced rather well by simulated results, generated with optimized coupling constants (spectra in Figure 4a,b; numerical data in Table 2).

**TABLE 2:** ESR Parameters (absolute values of coupling constants a, g factor, and linewidth) for some tetracene radical ions.

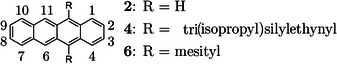							
	**4** 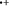 ^a^	**2** 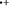 ^b^	**2** 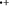 ^c^, ^d^	**6** 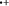 ^e^	**4** 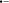 ^a^	**2** 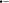 ^d^, ^f^	**2** 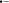 ^g^
$a_{6,11}$/G	3.98	5.06^h^	5.17^h^	5.0	2.78	4.25^h^	4.20^h^
$a_{1,4,7,10}$/G	1.58	1.69	1.72	1.85	1.25	1.55	1.48
	1.27			1.6	1.16		
$a_{2,3,8,9}$/G	0.61	1.03	1.06	1.0	0.76	1.15	1.21
	0.80			0.8	0.77		
g factor	2.0026	2.0026^i^, ^j^	2.0028^k^		2.0027	2.0027^j^, ^l^	
line width/G	0.16	0.15	0.055	0.85	0.080		

^a^ In 0.1 M ${\mathrm{NBu}}_{4}$ ${\mathrm{PF}}_{6}$/${\mathrm{CH}}_{2}$ ${\mathrm{Cl}}_{2}$, this work, electrochemical oxidation in situ.

^b^ In concentrated $H_{2}$ ${\mathrm{SO}}_{4}$ [65], chemical oxidation by solvent.

^c^ in 98 % sulphuric acid [66, 67].

^d^ slightly different values in refs. [68, 69]

^e^ in ${\mathrm{CH}}_{2}$ ${\mathrm{Cl}}_{2}$ [70], chemical oxidation by ${\mathrm{AgSbF}}_{6}$

^f^ in dimethoxyethane, reduction by sodium [66, 67]

^g^ in DMF [71], electrochemical reduction in external cell

^h^ this coupling constant is also valid for positions 5 and 12, which are substituted in the radical ions derived from **4** and **6**

^i^ in concentrated $H_{2}$ ${\mathrm{SO}}_{4}$, from refs. [72, 73]

^j^ value rounded to four decimal places, the original values use different types of corrections

^k^ from ref. [68]

^l^ from ref. [72].

According to this comparison between experimental and simulated spectra, only coupling with the aromatic protons in **4** is relevant. A hypothetical coupling with the Si nuclei in the TIPS substituents did not improve the fit significantly. The assignment of the coupling constants is based on a comparison with the radical cations of unsubstituted tetracene [65, 67, 69, 74] (created under various conditions), **2** 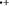, and a bis‐mesityl substituted derivative [70] **6** 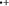. Thus, the largest a is assigned to the inner protons at positions 6 and 11 (for numbering of positions, see Table 2). Note that in the symmetric, unsubstituted radical cation **2** 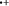 the largest coupling constant corresponds to 4 protons (positions 5, 6, 11, 12). The smaller coupling constants for **4** 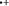 are assigned to the protons in the outer rings in two groups. This pattern is similar to that found in the ESR spectra of TIPS‐pentacene (**5** 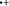) and pentacene (**3** 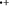) radical cations (see, refs. [36, 74] and references therein) and **6** 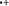. The g‐factor does not differ significantly from that of the unsubstituted parent and from that of the free electron. This again shows the negligible coupling with the Si atoms. The line width is in the same order of magnitude as reported for the tetracenes used as comparison.

### 2.3 Tetracene: Cyclic Voltammetry of First Oxidation Process

**FIGURE 5:**
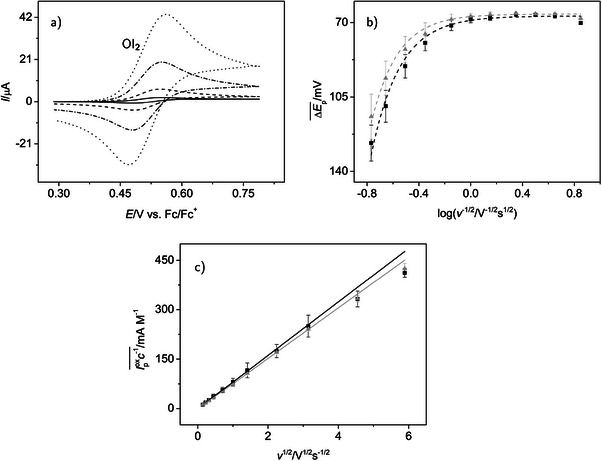
Cyclic voltammetry of tetracene **2** in the potential range of the first oxidation in ${\mathrm{NBu}}_{4}$ ${\mathrm{PF}}_{6}$/${\mathrm{CH}}_{2}$ ${\mathrm{Cl}}_{2}$ electrolytes (+0.288≤E/V≤+0.781); (a) cyclic voltammograms (c=0.19 mM, v=0.02, 0.2, 2.0, and 10 V $s^{-1}$ with increasing intensity of the signal), supporting electrolyte concentration: 0.1 M; (b) variation of peak potential difference with scan rate v, black squares: supporting electrolyte concentration 0.1 M, mean values of three independent measurements with 14 concentrations, 0.12≤c/mM≤0.22, gray triangles: supporting electrolyte concentration 0.2 M, mean values from four independent measurements with 19 concentrations, 0.13≤c/mM≤0.26; (c) variation of the concentration normalized oxidation peak currents $I_{p}^{\mathrm{ox}}$(${\mathrm{OI}}_{2})c^{-1}$ with $\sqrt{v}$, black squares and gray triangles as in (b), straight lines extrapolate the linear dependency found for small scan rates; the broken lines in (b) are only given as guide for the eye and do not carry physical meaning; error bars in (b) and (c) are standard deviations.

Similar to **4**, for a more quantitative investigation of the unsubstituted tetracene, **2**, we first restrict the potential range in cyclic voltammetry to the first oxidation process in the ${\mathrm{CH}}_{2}$ ${\mathrm{Cl}}_{2}$ electrolytes (Figure 5a) and extract the mo.st important features of the peaks observed. The peak potential difference at small v approaches the reversible limit of 58 mV for a one‐electron transfer process (Tables S2 and 3, entry 1), while $\Delta E_{p}$ increases with scan rates above about 1 V $s^{-1}$ (Tables S2 and 3, entry 2). As for the case of **4**, an increase with c(2) is almost negligible and only found at the highest scan rates used (Table S2). Thus, again, the experimental data are almost completely free of IR drop, thanks to the use of instrumental compensation (see Experimental Section). The shape of the voltammograms does not show any adsorption complications, which can also be derived from peak current analysis (see below). The formal potential of the electron transfer reaction is again available as the mid‐point potential. Only a negligible effect of the supporting electrolyte concentration on $E^{0}$(${\mathrm{OI}}_{2}$) can be observed (Table 3, entry 3).

**TABLE 3:** Cyclic voltammetric parameters characterizing first oxidation process of tetracene, ${\mathrm{OI}}_{2}$ (**2**/**2**).

	solvent c(${\mathrm{NBu}}_{4}$ ${\mathrm{PF}}_{6}$)/M	${\mathrm{CH}}_{2}$ ${\mathrm{Cl}}_{2}$		THF
entry		0.1	0.2	0.2
1	$\Delta E_{p}$(${\mathrm{OI}}_{2}$)/V^a^	0.067±0.001	0.066±0.001	—
2	$\Delta E_{p}$(${\mathrm{OI}}_{2}$)/V	0.109±0.008 ^b^	0.096±0.008 ^b^	0.132±0.008 ^c^
3	$E^{0}$(${\mathrm{OI}}_{2}$)/V^d^	0.518±0.001 ^e^	0.521±0.001 ^e^	0.553±0.018 ^c^
4	$I_{p}^{\mathrm{red}}\left({\text{OI}}_{2}\right)/I_{p}^{\mathrm{ox}}\left({\text{OI}}_{2}\right)$ ^f^	0.98±0.02 ^e^	0.98±0.02 ^e^	0.98±0.04 ^c^
5	$I_{p}^{\mathrm{red}}\left({\text{OI}}_{2}\right)/I_{p}^{\mathrm{ox}}$(${\mathrm{OI}}_{2}$)^f^	0.88±0.05 ^g^	0.87±0.07 ^g^	0.89±0.04 ^h^
6	$I_{p}^{\mathrm{ox}}\left({\text{OI}}_{2}\right)/\left(c\cdot \sqrt{v}\right)/$	2.39±0.02	2.40±0.03	2.15±0.09 ^i^
	mA $s^{1/2}$ $M^{-1}$ ${\mathrm{mV}}^{-1/2}$ ^e^			
7	$D{\left(2\right)}_{\mathrm{CV}}\cdot 10^{6}$/${\mathrm{cm}}^{2}$ $s^{-1}$ ^e^, ^j^	20.0±0.4	20.0±0.4	—
8	$k_{s}$(${\mathrm{OI}}_{2}$)/cm $s^{-1}$ ^k^	0.09±0.02	0.13±0.05	—

^a^ with close to fully reversible shape of voltammograms, for small scan rates: 0.05≤v/ V $s^{-1}\leq 1.0$, mean values over all concentrations of all experiments.

^b^ for 20 V $s^{-1}$.

^c^ for 36 V $s^{-1}$.

^d^ determined as midpoint potential.

^e^ for 0.1≤v/ V $s^{-1}\leq 1.0$.

^f^ calculated with Nicholson's equation [59].

^g^ for v=0.05 V $s^{-1}$.

^h^ for 21 V $s^{-1}$.

^i^ for 10≤v/ V $s^{-1}\leq 35$; three independent experiments with 0.14 ≤ *c*/mM ≤ 0.26.

^j^ from oxidation peak currents according to Randles–Ševčík equation in the form given by Nicholson and Shain [60+].

^k^ from peak potential differences at high scan rates based on ref. [61+], for v≥10 V $s^{-1}$.

The peak current ratio (Table 3, entries 4 and 5) is close to unity at slow to medium scan rates, while it significantly decreases at the smaller v=0.05 V $s^{-1}$ for the same reasons as given for **4** above. The peak current function, as averaged over the respective v up to 1 V $s^{-1}$ and all c(2) is constant (Table 3, entry 6).

The diffusion coefficients calculated from the peak currents (Table 3, entry 7) are considerably higher than those of TIPS‐tetracene, and the effect of the supporting electrolyte concentration has disappeared. The increase in D when going from **4** to **2** is reasonable owing to the decrease in molecular weight and the smaller size of the unsubsituted molecule. We note that these results could be somewhat hampered by the lagging dissolution of **2** (see Experimental Section, Electrochemical Data Generation and Treatment).

With knowledge of the diffusion coefficient, the peak potential differences determined at *large* scan rates (quasi‐reversible range; Figure 5b) provide rate constants of the heterogeneous electron transfer of **2** (entry 8 in Table 3). At these larger scan rates, the concentration normalized peak currents (Figure 5c; values plotted vs. the square root of v) deviate to smaller values from the straight Randles–Ševčík type line, which is valid at small scan rates. This excludes significant effects of adsorption, where the normalized peak currents would be expected to lie above the straight line, because i becomes proportional to v [75]. Compared to **4**, the rate constant for **2** is somewhat higher, possibly indicating a steric effect of the bulky substituents in **4**. Note that in accordance with the fact that D(2) is independent of the supporting electrolyte concentration, there remains only a small effect of the electrolyte composition on $\Delta E_{p}$ (Figure 5b) and differences in $I_{p}^{\mathrm{ox}}/c$ with c(${\mathrm{NBu}}_{4}$ ${\mathrm{PF}}_{6}$) have essentially disappeared (Figure 5c).

**FIGURE 6:**
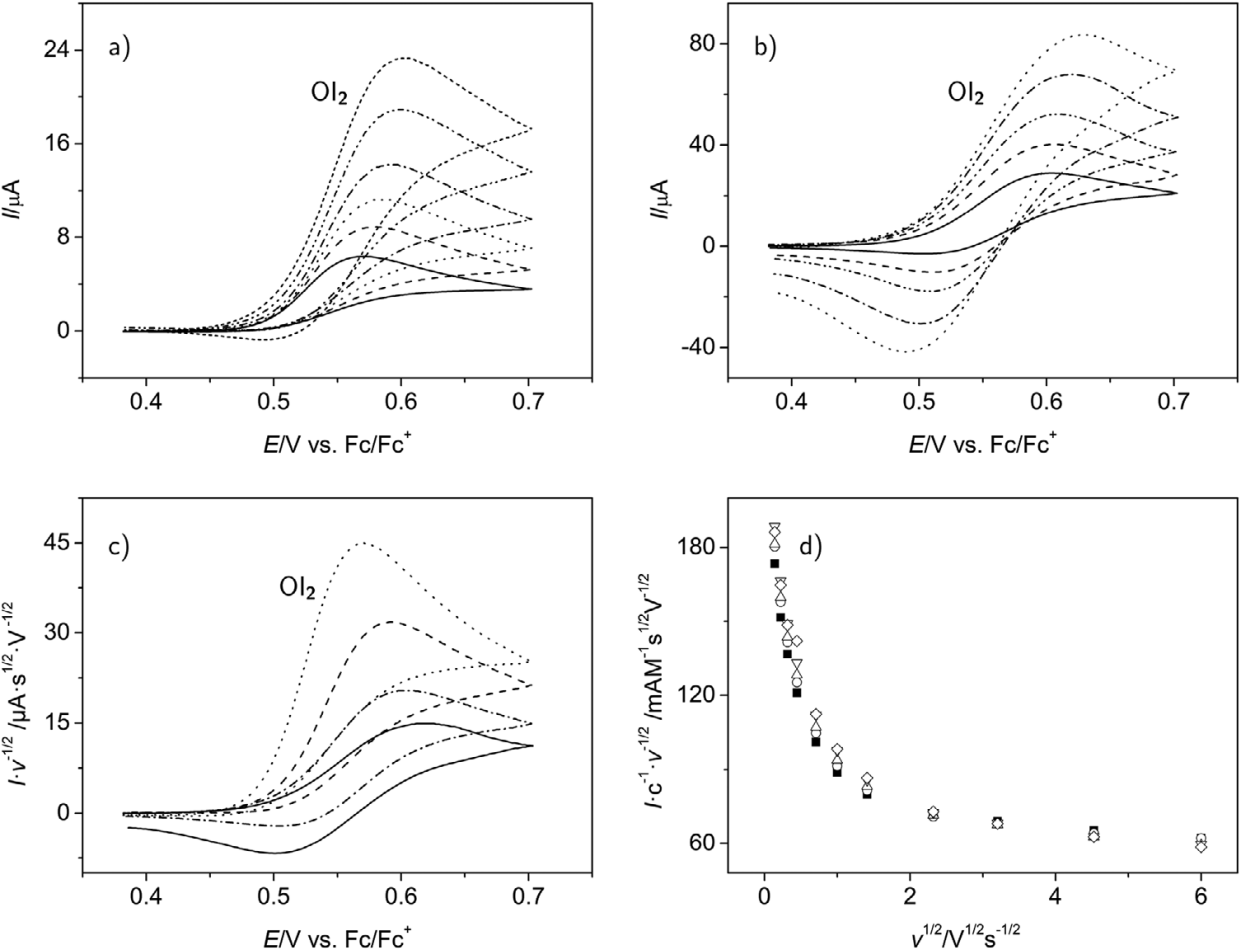
Cyclic voltammetry of tetracene **2** in the potential range of the first oxidation in a 0.2 M ${\mathrm{NBu}}_{4}$ ${\mathrm{PF}}_{6}$/THF electrolyte (+0.382≤E/V≤+0.702); (a) cyclic voltammograms (c=0.24 mM, v=0.02, 0.2, 0.5, and 1.0 V $s^{-1}$ with increasing intensity of the signal); (b) same as (a), but for v=2.0, 5.0, 10, 21, and 36 V $s^{-1}$; (c) scan rate normalized voltammograms (c=0.24 mM, v=0.02, 0.2, 2.0, and 21 V $s^{-1}$, with decreasing intensity); (d) scan rate normalized oxidation peak currents, 0.02≤v/ V $s^{-1}$ ≤36, concentrations of **2**: 0.14 mM (filled squares), 0.18 mM (circles), 0.22 mM (upward pointing triangles), 0.24 mM (downward pointing triangles), 0.26 mM (open squares).

In the THF‐based electrolyte with 0.2 M ${\mathrm{NBu}}_{4}$ ${\mathrm{PF}}_{6}$ as the supporting electrolyte, more complex effects of follow‐up reactions of the one‐electron oxidation product of **2** become obvious (see Figure 6 and numerical values in Table 3, data column 3). As already shown by the overview voltammogram of **2** in the THF electrolyte with a relatively small scan rate (Figure 2b), the reverse peak of process ${\mathrm{OI}}_{2}$ is absent for slower time scales. Scan rate dependent cyclic voltammetry indicates that this reverse peak starts to appear at v≥≈1 V $s^{-1}$. It reaches its full extent at our upper scan rate limit (v=36 V $s^{-1}$; Figure 6a,b). The scan rate dependent values of the peak current ratio for this process support the conclusion of a follow‐up chemical reaction that consumes the primary oxidation product created in the oxidation reaction. Only at the upper v limit, the ratio approaches a value of unity, and thus indicates chemical reversibility (Table 3, data column 3, entries 4 and 5). Further support is provided by the shift of the peak potential of the oxidation peak to more positive values upon an increase in v, as indicated in Figure 6a. This behavior is characteristic for a mechanism with a follow‐up reaction coupled to the electron transfer [60+]. In addition, the peak potential difference increases at larger scan rates (Figure 6b). Thus, while the follow‐up reaction looses importance with increasing v, the electron transfer enters the quasi‐reversible region under these conditions (see $\Delta E_{p}$ at v=36 V $s^{-1}$, Table 3, data column 3, entry 2).

Since none of the experimental conditions allows the observation of fully reversible voltammogram shapes, it is difficult to make a direct conclusion about the number of transferred electrons. However, it seems highly likely that again we deal with a one‐electron process as in the case of the first oxidation process of **4**. This is indicated by a comparison of the concentration and scan rate normalized peak currents for peaks ${\mathrm{OI}}_{2}^{\mathrm{ox}}$ (Table 3) and ${\mathrm{RI}}_{2}^{\mathrm{red}}$ (Table 4). The reduction process will be shown below to involve one electron. Of course, the exact value of the theoretical peak current function for quasi‐reversible peaks (${\mathrm{OI}}_{2}$) is somewhat smaller than that for a fully reversible peak (${\mathrm{RI}}_{2}$). However, for the present conditions, the difference is expected to be small [61+].

**TABLE 4:** Cyclic voltammetric parameters characterizing reduction processes of TIPS‐tetracene **4** and tetracene **2** in THF and ${\mathrm{CH}}_{2}$ ${\mathrm{Cl}}_{2}$ electrolytes.^a^

	solvent c(${\mathrm{NBu}}_{4}$ ${\mathrm{PF}}_{6}$) redox couple(s)	${\mathrm{CH}}_{2}$ ${\mathrm{Cl}}_{2}$		THF	
		0.1 M		0.2 M	
entry		**2**/**2** 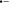	**4**/**4** 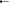 /**4** 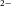	**2**/**2** 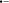 /**2** 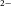	**4**/**4** 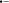 /**4** 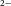
1	$\Delta E_{p}$(RI)	0.072 ± 0.003	0.069 ± 0.002	0.068 ± 0.002	0.070 ± 0.003
2	$E^{0}$(RI)/V	−2.102±0.001	−1.689±0.002	−2.086±0.001	−1.643±0.003
3	$E^{0}$(RII)/V	− ^b^	−2.240 ^c^	−2.638 ^c^	−2.174±0.002
4	$I_{p}^{\mathrm{red}}\left(\text{RI}\right)/\left(c\sqrt{v}\right)/$	2.52±0.04	1.65±0.07	2.12±0.05	1.30±0.04
	mA $s^{1/2}$ $M^{-1}$ ${\mathrm{mV}}^{-1/2}$				

^a^ mean values from experiments with $0.05\leq v/V\;s^{-1}\leq 0.5$ and 0.47≤c/mM≤0.78.

^b^ only one reduction signal inside potential window.

^c^ determined as half wave potential of the reduction peak.

Still, at the large scan rates, where chemical reversibility is attained, the midpoint potential will provide a good estimate of the formal potential of the first oxidation of **2** in the THF electrolyte (Table 3, data column 3, entry 3). Compared to the situation in the ${\mathrm{CH}}_{2}$ ${\mathrm{Cl}}_{2}$ electrolytes discussed before, $E^{0}$(${\mathrm{OI}}_{2}$) attains a slightly more positive value in THF. However, the difference is only ≈30 mV, much less than the difference in the case of **4**. Note that recently a value of 0.503 V was reported for **2** in 1,2,3,4‐tetrafluorobenzene with a supporting electrolyte based on a weakly coordinating anion [74]. This compares well with our results in ${\mathrm{CH}}_{2}$ ${\mathrm{Cl}}_{2}$ and THF. We conclude that the solvent effect on $E^{0}$(${\mathrm{OI}}_{2}$) is much smaller than that found for TIPS‐tetracene.

A plot of current–potential curves, which are normalized by the square root of the scan rate (Figure 6c), indicates that the follow‐up reaction must be more complex than a simple irreversible reaction step. In the latter case, we would expect only a slight increase in the normalized peak currents upon a decrease of v [60+]. In contrast, in the case of oxidation process ${\mathrm{OI}}_{2}$, these peak currents increase (at the low v limit) to more than three times the value found at the high scan rate limit (Figure 6d). This behavior shows that additional electron transfers are induced by the decay of the primary oxidation product. As a result, an ECE‐type mechanism (sequence of electrochemical–chemical–electrochemical steps) must be assumed, where the additional steps even include more than one electron. It is also noted that the effect of the chemical reaction step is independent of c(2) (Figure 6d), indicating only first‐order kinetic contributions.

Although being produced by a one‐electron oxidation in both ${\mathrm{CH}}_{2}$ ${\mathrm{Cl}}_{2}$ and THF similar to the case of **4**, the radical cation of **2** without the TIPS substituents becomes unstable in THF and undergoes a chemical reaction, which induces further electron transfers resulting in additional current contributions. This observation clearly substantiates the notion of acene radical cation stabilization by the protecting TIPS substituents with respect to chemical reaction at least in THF.

### 2.4 Reduction Processes of Tetracene and TIPS‐Tetracene

Cyclic voltammetric scans in the potential region negative of the rest potential at various concentrations in the millimolar range and (if experimentally feasible without electrode fouling) a number of scan rates yield information about reduction processes of **2** and **4** in the electrolytes used in this work. For both compounds, under all electrolyte conditions a primary one‐electron reduction is apparent, which is electrochemically reversible, as shown by the peak potential differences $\Delta E_{p}$(RI) being close to the reversible limit for n=1 (see Table 4, first entry). Only at very low scan rates (v=0.02 V $s^{-1}$) the reduction process of **2** in 0.1 M ${\mathrm{NBu}}_{4}$ ${\mathrm{PF}}_{6}$/${\mathrm{CH}}_{2}$ ${\mathrm{Cl}}_{2}$ becomes chemically irreversible with a chemical reaction of the radical anion product as indicated by the absence of a reverse peak (Figure S2). For **2** in ${\mathrm{CH}}_{2}$ ${\mathrm{Cl}}_{2}$ and **4** in both electrolytes with 0.1 M ${\mathrm{NBu}}_{4}$ ${\mathrm{PF}}_{6}$, the peak current $I_{p}^{\mathrm{red}}$(RI) is approximately equal to the respective $I_{p}^{\mathrm{ox}}$(OI) (see the peak intensities in Figure 2). This is expected if both, OI and RI, are processes involving the same number of electrons, here n=1. The relative variation of $I_{p}^{\mathrm{red}}\left(\mathrm{RI}\right)/\left(c\sqrt{v}\right)$ (Table 4, entry 4) with the solvent and substitution is similar to the corresponding normalized currents for one‐electron process OI. The formal potentials of the primary reductions were estimated as the mid‐point potentials in all cases and are listed in Table 4 as entry 2.

In the case of **4**, we identified the primary reduction product by in situ electrolysis at constant cell voltage inside the cavity of an ESR spectrometer. The experimental spectrum was matched by a simulation (Figure 4c,d, coupling constants in Table 2). Similar to the radical cation case, the coupling constants in the radical anion **4** 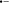 decrease going from positions 6/11 to the edge of the polycyclic aromatic system, as has been also observed for radical anion **2** 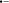 [66, 67, 71].

A second reduction peak is found within the potential window for **4** in electrolytes based both on ${\mathrm{CH}}_{2}$ ${\mathrm{Cl}}_{2}$ and THF, but for **2** this process seems only accessible in THF and shows characteristics of a chemical follow‐up reaction (missing reverse peak). The reduction peak current $I_{p}^{\mathrm{red}}$(${\mathrm{RII}}_{4}$) in THF is again approximately equal to $I_{p}^{\mathrm{ox}}$(${\mathrm{OI}}_{4}$) and it follows again n=1. TIPS‐tetracene is reduced in THF solution in a stepwise EE mechanism (two separate electrochemical, i.e., electron transfer, steps) with two electrons and normal potential ordering [76] and stability of all redox states in the time scale of the voltammograms (e.g., v=0.2 V $s^{-1}$ in Figure 2d). In ${\mathrm{CH}}_{2}$ ${\mathrm{Cl}}_{2}$, ${\mathrm{RII}}_{4}$ is located close to the negative limit of the potential window, which prohibits more quantitative analysis of its behavior. Owing to the fact that the second reduction of **4** in ${\mathrm{CH}}_{2}$ ${\mathrm{Cl}}_{2}$ is associated with a chemical follow‐up reaction as indicated by the missing reverse oxidation peak and the occurrence of ${\mathrm{OIII}}_{4}$ (see the discussion of this peak above) as well as the observation that peak ${\mathrm{RII}}_{4}^{\mathrm{red}}$ has an unusual flat shape, we only give a value of the half wave potential (current reaches 50 % of its peak value; Table 4, entry 3, second data column). Note that this value is probably shifted to more positive values from $E^{0}$(${\mathrm{RII}}_{4}$) by the kinetics of the coupled chemical step and is not necessarily comparable to the formal potentials given for the other redox processes. Also, for the irreversible reduction peak ${\mathrm{RII}}_{2}^{\mathrm{red}}$ in THF only the half wave potential is given.

### 2.5 Second Oxidation Processes of Tetracene and TIPS‐Tetracene

In the ${\mathrm{CH}}_{2}$ ${\mathrm{Cl}}_{2}$ electrolytes, a second oxidation process OII is evident for both tetracene and TIPS‐tetracene in the cyclic voltammograms and located close to the positive limit of the accessible potentials (Figure 2a,c). More detailed voltammetric data and their interpretation are presented in the Supporting Information.

The features of the second oxidation processes, which lead to the oxidation state of dications, indicate irreversible chemical follow‐up reactions. Only for the substituted **4** we observe some incipient stabilization of the dication at high scan rates. The cyclic voltammetric pattern is similar to TIPS‐pentacene **5**, but the coupled chemical reactions are faster in the two tetracenes investigated here.

### 2.6 Computational Results

According to DFT calculations as detailed in the Supporting Information (see also numerical results in Table S5), the gas phase ionization energy (IE) of **4** (6.31 eV) is smaller than that of **2** (6.65 eV). In a ${\mathrm{CH}}_{2}$ ${\mathrm{Cl}}_{2}$ environment, however, the IE of **4** (5.14 eV) is larger than that of **2** (5.12 eV). The same is true for THF, where IE of **4** is 5.18 eV and that of **2** is 5.17 eV. Within computational accuracy, it is safe to say that the IE of both acenes are identical within a given solvent system. The reason for the essentially identical IE in the solvents is rooted in the difference of free energies of solvation $\Delta \delta G_{\mathrm{solv}}$. In particular, the radical cations **2** 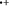 and **4** 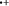 appear to have very similar $\delta G_{\mathrm{solv}}$ to within 0.04 eV in ${\mathrm{CH}}_{2}$ ${\mathrm{Cl}}_{2}$ and 0.06 eV in THF. This is not the case for the neutrals, where **4** is experiencing more stabilization due to solvation than **2** by 0.40 eV (${\mathrm{CH}}_{2}$ ${\mathrm{Cl}}_{2}$) and 0.28 eV (THF). The main effect of the environmental change from the gas to the solution phase must be associated with the stabilization of the neutral compounds.

On the other hand, the electron affinities (EA) of **4** and **2** differ by 0.75 eV in the gas phase and by 0.53 eV (${\mathrm{CH}}_{2}$ ${\mathrm{Cl}}_{2}$) or 0.52 eV (THF) in solution. Radical anion **4** 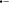 profits less from solvation than the neutral by about 0.2 eV, causing the reduced differences in EA compared to the gas phase.

## 3 Comparative Discussion

Tetracene **2** and TIPS‐tetracene **4** show some common patterns in the cyclic voltammograms. The behavior is, however, modulated by the accessible potential window in the solvent used as the base for the electrolyte solution and the effect of the TIPS substituents through the resulting shift of formal potentials with respect to Fc/${\mathrm{Fc}}^{+}$. The overall reduction and oxidation mechanisms of both **2** and **4** involve two electrons under at least some conditions. While the primary (first) oxidation and reduction processes of both **2** and **4** could be observed in all experiments, the second redox processes are outside of the accessible potential range at least under some conditions. The second oxidation process is evident in the ${\mathrm{CH}}_{2}$ ${\mathrm{Cl}}_{2}$ electrolytes, but absent in 0.2 M ${\mathrm{NBu}}_{4}$ ${\mathrm{PF}}_{6}$/THF. The second reduction process is absent for **2** in ${\mathrm{CH}}_{2}$ ${\mathrm{Cl}}_{2}$, but appears in all other combinations. Only for TIPS‐tetracene in a ${\mathrm{CH}}_{2}$ ${\mathrm{Cl}}_{2}$ electrolyte, all four redox processes show up. Then, we also find an additional signal of the reduction product, which is probably caused by chemical reaction with the solvent, when scanning through the potential range of the second reduction process (see discussion of overview voltammograms above).

In the cyclic voltammetric experiments, we could determine formal redox potentials $E^{0}$ under chemically and electrochemically reversible conditions by restricting the analyses to certain time scales, if necessary. Kinetic effects (related to both, chemical reactions and the electron transfer) were thus eliminated. The data were generated for various concentrations and (in most cases) also various scan rates (time scales). The $E^{0}$ results are mean values over a large number of individual experiments.

**FIGURE 7:**
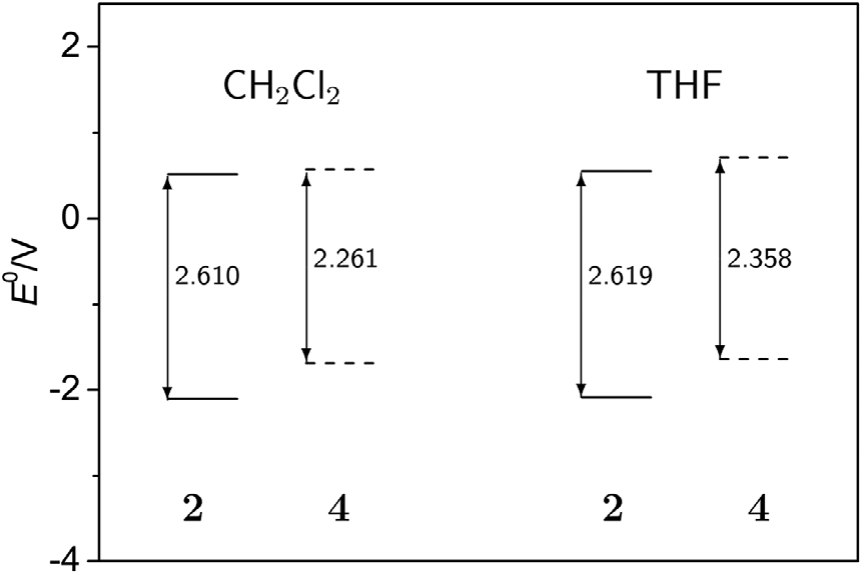
Formal potentials for first oxidation and first reduction of **2** and **4**, leading to the respective radical ions, in 0.1 M ${\mathrm{NBu}}_{4}$ ${\mathrm{PF}}_{6}$/${\mathrm{CH}}_{2}$ ${\mathrm{Cl}}_{2}$ and 0.2 M ${\mathrm{NBu}}_{4}$ ${\mathrm{PF}}_{6}$/THF electrolytes with values of the electrochemical gap $\Delta E^{0}$/V.

Redox potentials are sometimes discussed in the context of ionization energies. Gas phase ionisation energies of TIPS‐substituted acenes are lower compared to those of the unsubstituted parent acenes **1** 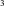, **1** 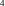, and **1** 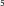 [39, 77]. The tri(isopropyl)silylethynyl group exerts an electron donating substituent effect under such conditions. This is confirmed by the current DFT calculations (Table S5). In electrochemical experiments of **2** and **4**, this substituent effect should result in a lower $E^{0}$(OI) for the substituted tetracene. However, the potential shift for the first oxidation process is opposite, that is, TIPS‐tetracene **4** is oxidized at a more positive potential than unsubstituted tetracene **2** in both ${\mathrm{CH}}_{2}$ ${\mathrm{Cl}}_{2}$ and THF (Figure 7 and Tables 1 and 3). For the couple pentacene/TIPS‐pentacene, a similar pattern was described (electrochemistry in *ortho*‐dichlorobenzene as a solvent) and explained through a size effect and polarization by the surrounding solvent molecules [33]. Such effects are absent in the gas phase. HOMO energies were “reported to ±0.05 eV” [33] (converted from electrochemical potentials in *ortho*‐dichlorobenzene) with identical values for unsubstituted **3** and substituted **5**. The “similarity of redox potentials” was noted [33]. However, a small shift of $E^{0}$ may have gone undetected based on this large uncertainty in the measured potential data.

The ionization energies for **2** and **4** in both solvent environments are smaller—according to our calculations—than those in the gas phase, but the substituent effect has essentially vanished (see paragraph Computational Results and Table S5). Compared to vacuum conditions, the liquid electrolyte environment seems to stabilize the unsubstituted acene radical cation more or stabilize the respective neutral starting compound to a lesser degree than the substituted one. As mentioned in Section “Computational Results”, the main solvation effect is the stabilization of the neutral acenes.

In the present work, we report potentials in the electrochemical experiments with respect to the Fc/${\mathrm{Fc}}^{+}$ reference, which is conventionally assumed to be solvent independent [78]. It is thus possible to compare the formal potentials in the two solvents used for our electrolytes. The direction of the redox potential shift caused by the substituents is the same for both solvents. However, it is stronger in the THF‐ (0.162 V) as compared to the ${\mathrm{CH}}_{2}$ ${\mathrm{Cl}}_{2}$‐based (≈0.05 V) electrolyte.

Since the solvent effect on $E^{0}$(${\mathrm{OI}}_{2}$) when changing from ${\mathrm{CH}}_{2}$ ${\mathrm{Cl}}_{2}$ to THF (≈0.03 V) is much smaller than that on $E^{0}$(${\mathrm{OI}}_{4}$) (≈0.14 V), our electrochemical data support the explanation by interactions between the substituent and the solvent. On the other hand, the solvent effect displayed by the calculated ionization energies is very small (0.05 eV for **2** and 0.04 eV for **4**). Given the complex factors that contribute to the relative energies of the acenes and their radical cations, we will not attempt to further interpret this result here. However, it appears that the electrochemical data include effects that are not considered in the calculations (interactions with supporting electrolyte ions or inner solvation shell, double layer effects).

For the reduction processes RI, the solvent effect is small (<0.05 V) but still significant for both **2** and **4**. Although it is again smaller for the unsubstituted compound, as we found for OI, its absolute value reaches only 50% or less compared to the oxidation process. A weak dependency of reduction half‐wave potentials on the solvent has been noted before for benzenoid hydrocarbons more generally [79]. On the other hand, the substituent effect on $E^{0}$(RI) is >0.4 V, much larger than for $E^{0}$(OI), with the substituted compound **4** being more easily reduced. In parallel, and in contrast to the ionization energy, the electron affinity of **4** in solution is considerably more negative than that of **2** (see paragraph Computational Results and Table S5) for both electrolytes.

Similar to the case of OI, the $E^{0}$(RI) in the THF electrolyte are more affected by the substituent as compared to ${\mathrm{CH}}_{2}$ ${\mathrm{Cl}}_{2}$. However, the substituent effect does vary much less between the solvents for process RI. Since this is not reflected in the calculation results for the electron affinities, where the effect of the solvent for both **2** and **4** is in the same range as that for the ionization energies, we must again assume that the electrochemical data are controlled by additional factors.

It has been mentioned several times in earlier work that the LUMO energies of acenes are much more [39, 80, 81, 82] dependent on the substitution than the HOMO energies [33, 80, 81, 82]. It is also commonly argued that the frontier orbital energies are related to the redox potentials by an “excellent correlation” between experimental electrochemical potentials and calculated orbital energies [79]. For example, correlations were found for electron affinities or Hückel LUMO energies and polarographic reduction potentials of aromatic hydrocarbons [83]. There are, however, fundamental and practical problems that prohibit direct conversion between redox potentials and orbital energies [45, 84, 85]. The following discussion will thus only be in terms of the redox potentials, which should *not* directly be interpreted as frontier orbital energies.

The different effect on the $E^{0}$ for the first oxidation and reduction processes is also seen in our electrochemical results based on (except for **4** in THF) scan rate and concentration‐dependent experiments and with a high potential resolution for tetracene/TIPS‐tetracene in both solvents (Figure 7). As a general rule, the TIPS substituent renders the acenes more difficult to oxidize, but more easy to reduce. It thus appears to decrease the electron density, and for electrochemical conditions (in solution, with supporting electrolyte ions present), the tri(isopropyl)silylethynyl substituent has an electron‐withdrawing effect. The direction of the effect is independent of, but its extent is modified by, the solvent.

From our results for the formal potentials, values for an “electrochemical gap” $\Delta E^{0}=E^{0}\left(\mathrm{OI}\right)-E^{0}\left(\mathrm{RI}\right)$ were calculated and are included in Figure 7. We used the ${\mathrm{CH}}_{2}$ ${\mathrm{Cl}}_{2}$ data with 0.1 M supporting electrolyte concentration based on the more complete data basis. As shown above (Tables 1 and 3), the effect of c(${\mathrm{NBu}}_{4}$ ${\mathrm{PF}}_{6}$) on $E^{0}$(OI) is negligible.

The substituent effect on $\Delta E^{0}$ is approximately 0.25 V (THF) and 0.35 V (${\mathrm{CH}}_{2}$ ${\mathrm{Cl}}_{2}$) with most of the difference attributed to the effect on the value of $E^{0}$(RI). While the solvent effect on $\Delta E^{0}$(**2**) is only a few mV, it is considerably higher (≈0.1 V) for the substituted compound **4**. While we lack similar data for the unsubstituted pentacene **1** 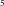, we can compare the values calculated in the present work with those for TIPS‐pentacene **5**. The formal potentials for the first reduction of **5** are −1.480 in 0.1 M ${\mathrm{NBu}}_{4}$ ${\mathrm{PF}}_{6}$/${\mathrm{CH}}_{2}$ ${\mathrm{Cl}}_{2}$ and −1.427 V in 0.2 M ${\mathrm{NBu}}_{4}$ ${\mathrm{PF}}_{6}$/THF. Values for $E^{0}$(${\mathrm{OI}}_{5}$) were reported in ref. [36], resulting in electrochemical gaps of 1.847 V in the ${\mathrm{CH}}_{2}$ ${\mathrm{Cl}}_{2}$ and 1.949 V in the THF electrolyte. While the solvent effect is consistent in size and direction with what we observe for **4**, the absolute values of $\Delta E^{0}$ are smaller for the more extended pentacene derivative, in accordance with the increased size of the π‐system, a trend described earlier for 9,10‐bis(tri(isopropyl)silylethynyl)anthracene, TIPS‐tetracene, and TIPS‐pentacene for differential pulse voltammetric data in ${\mathrm{CH}}_{2}$ ${\mathrm{Cl}}_{2}$ [39].

For all compounds discussed in the present paper, but particularly for the TIPS‐derivatives **4** and **5**, the exact values of the electrochemical gap depend on the solvent environment. This shows the importance to clearly separate the concepts of redox potential and frontier orbital energy and the resulting advice to not simply estimate such energies or the HOMO–LUMO gap from electrochemical data. Rather, interactions of the redox species with the environment modulate the $E^{0}$ and their difference [45]. Although the effects on $E^{0}$ or $\Delta E^{0}$ may correlate to a certain degree with those on individual frontier orbital energies and on HOMO–LUMO gaps derived by calculation or from photophysical experiments, the electrochemical gap should be considered as a molecular property of its own right in a certain electrolyte environment, and values should be clearly labeled as such. As the example of **2** and **4** shows, $\Delta E^{0}$ depends on a complex combination of substituent (intrinsic) and solvent (extrinsic) effects. Of course, this does not preclude parallels to the influence on the frontier orbital energies or on the HOMO–LUMO gap.

In the THF electrolyte, we find conditions that allow to demonstrate the effect of the TIPS‐substituents with respect to chemical reactivity. Chemical decay of the acene radical cation is suggested by the peak current ratios $I_{p}^{\mathrm{red}}$(OI)/$I_{p}^{\mathrm{ox}}$(OI) for **2** and **4**. While this ratio is close to the value of unity (absence of a follow‐up reaction) in the case of **4** for scan rates down to 0.05 V $s^{-1}$, considerably faster time scales (v=21 ${\mathrm{Vs}}^{-1}$) are necessary to eliminate the kinetic effects in the case of **2**. Thus, the TIPS substituents decrease the rate of radical cation decay in THF by a factor of approximately 400. A similar effect on the reactivity in ${\mathrm{CH}}_{2}$ ${\mathrm{Cl}}_{2}$ is much smaller than in THF (see peak current ratios for **2** and **4** at v=0.05 V $s^{-1}$ in Tables 1 and 3). Note that we recently unveiled an electrochemically induced (4+2)‐cycloaddition of the TIPS‐pentacene radical cation **5** 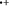 on a time scale much slower than achievable under cyclic voltammetric conditions, but characteristic for bulk electrolysis experiments (extending over several hours) [44]. This reaction involved the ethynyl moiety of one TIPS substituent.

## 4 Conclusions

Formal redox potentials $E^{0}$ of tetracene **2** and TIPS‐tetracene **4** have been determined from cyclic voltammograms under strictly electrochemically and chemically reversible conditions in two solvents, ${\mathrm{CH}}_{2}$ ${\mathrm{Cl}}_{2}$ and THF. Kinetic modulation of the data is avoided. The tri(isopropyl)silylethynyl substituents show an electron‐withdrawing effect in the electrochemical experiment in both solvents, in contrast to the electron‐donating effect concluded from gas‐phase ionization energies in the literature. The influence of the substituent is seen in oxidation *and* reduction formal potentials, but the effect is more pronounced for the reduction. The effect of the solvent on $E^{0}$ is much larger in the TIPS‐tetracene, indicating involvement of the substituent in the interaction with the solvent.

The substituent effect is also apparent with respect to the “electrochemical gap” $\Delta E^{0}$, with the influence of the solvent on $\Delta E^{0}$(**4**) being much larger than that on $\Delta E^{0}$(**2**). Based on our results, we suggest that $\Delta E^{0}$ should be reported as an extrinsically modulated molecular property. Comparison with gas phase results and calculated HOMO–LUMO gaps should only be done considering the different environments.

Time scale‐dependent analysis for the not fully reversible processes yields kinetic ($k_{s}$) and mechanistic information about electron transfers and/or coupled chemical reactions. In this context, the electrochemical results also highlight the stabilizing effect of the tri(isopropyl)silylethynyl substituent by slowing down the chemical kinetics of radical cation decay on the voltammetric time scale, while our previous study [44] has shown involvement of this substituent in a pentacene derivate leading to additional cycloaddition reactivity.

Thus, TIPS as a substituent and the solvent do not only shift redox potentials, but also modulate the reactivity of the radical cations. In particular, we note that the widely used TIPS substituent, often regarded as a “protecting group”, exhibits a complex behavior with respect to chemical reactivity in acenes: on one hand, it will stabilize the overall molecule, but on the other hand, it might take part in additional reaction paths.

## 5 Experimental Section

### 5.1 Chemicals

${\mathrm{AgClO}}_{4}$, ${\mathrm{NBu}}_{4}$ ${\mathrm{PF}}_{6}$, ferrocene (Alfa–Aesar), and other chemicals were of reagent grade, if not otherwise mentioned. Tetracene (TCI) was of 98% purity. TIPS‐Tetracene was synthesized as described previously [25]. All chemicals, as well as dichloromethane‐$d_{2}$ (Sigma–Aldrich) and THF‐$d_{8}$ (euriso‐top), were used as received, except if noted otherwise below.

Dichloromethane (Th. Geyer, stabilized with ethanol) and acetonitrile (J.T. Baker) were of HPLC grade and purified for electrochemical purposes as described below.

### 5.2 Electrochemical Experiments

#### 5.2.1 General

Dichloromethane was first distilled over $P_{2}$ $O_{5}$ and then over $K_{2}$ ${\mathrm{CO}}_{3}$. The purified solvent was stored over basic ${\mathrm{Al}}_{2}$ $O_{3}$ (activated at 240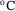 under reduced pressure for 3 days) and under argon. It was used within 1 week without noticeable degradation. Acetonitrile was distilled successively over $P_{2}$ $O_{5}$, ${\mathrm{CaH}}_{2}$, and $P_{2}$ $O_{5}$ again. Purified acetonitrile was stored over molecular sieves (3 Å, activated at 170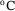 under reduced pressure for 3 days).

${\mathrm{NBu}}_{4}$ ${\mathrm{PF}}_{6}$ was recrystallized four times from ethanol/water 3:1 and dried at ≈3 mbar and 105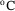 for 1 week.

As supporting electrolyte, ${\mathrm{NBu}}_{4}$ ${\mathrm{PF}}_{6}$ was used in a concentration of 0.1 M. After dissolving the salt, the electrolyte was degassed by freeze–pump–thaw cycles before it was transferred to the cell.

#### 5.2.2 Electroanalytical Equipment

Electroanalytical experiments were performed at 17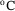 under an argon atmosphere with an ECO‐Autolab PGSTAT100 (Metrohm) with GPES‐Software 4.9.007.

The electrochemical cell, including the electrodes, for cyclic voltammetry was already described before [36, 44].

#### 5.2.3 Reference Potentials

Potential values in this work are rescaled to an external Fc/${\mathrm{Fc}}^{+}$ reference [78], determined in the same cell as described above (mean values vs. Ag/${\mathrm{Ag}}^{+}$: $E^{0}$ (Fc/${\mathrm{Fc}}^{+}$) =+0.213 V for 0.1 M ${\mathrm{NBu}}_{4}$ ${\mathrm{PF}}_{6}$/${\mathrm{CH}}_{2}$ ${\mathrm{Cl}}_{2}$; $E^{0}$ (Fc/${\mathrm{Fc}}^{+}$) =+0.199 V for 0.2 M ${\mathrm{NBu}}_{4}$ ${\mathrm{PF}}_{6}$/${\mathrm{CH}}_{2}$ ${\mathrm{Cl}}_{2}$; $E^{0}$ (Fc/${\mathrm{Fc}}^{+}$) =+0.162 V for 0.2 M ${\mathrm{NBu}}_{4}$ ${\mathrm{PF}}_{6}$/THF) in regular intervals and in close proximity in time to the experiments.

#### 5.2.4 Electrochemical Data Generation and Treatment

IR drop was compensated by positive feedback under control of the GPES software. Background cyclic voltammograms were recorded for various scan rates at the beginning of an experiment session for later use. Then, substrate was added (if applicable in several aliquots) from stock solutions by a gas‐tight syringe (Hamilton). In the case of unsubstituted tetracene, dissolution of the sample in the electrolyte had to be supported by ultrasonication. Cyclic voltammograms were recorded at the same set of scan rates after each addition, and the respective background currents were subtracted from the resulting data. Interfering high frequency current signals were filtered using the PGSTAT 100 instrument's “smooth” function (algorithm: Fast Fourier transform).

All voltammograms were recorded with a step width of 0.001 V.

Heterogeneous electron‐transfer rate constants, $k_{s}$, were estimated by comparing the scan rate dependence of experimental peak potential differences $\Delta E_{p}$ to simulated values in analogy to Nicholson's working curve approach [61+], but using simulation data for a temperature of 17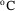. Thus, for each experimental $\Delta E_{p}$, the corresponding value of ψ was found from the nonlinear working curve. Since $\psi =\sqrt{RT/\pi DnFv}k_{s}$, from each ψ follows a value for $k_{s}$, if D and v are known. The number of transferred electrons was set to n=1.

### 5.3 Spectroscopy

*X‐band ESR spectroelectrochemical experiments* on an ESP 300E spectrometer (Bruker) were conducted at room temperature in analogy to the procedure described in ref. [36]. The optimized terminal voltage U to produce the respective radical cation is given in the legend to Figures 4.

g factors were determined relative to “strong pitch” (g=2.0028) [86]. For simulations of the ESR spectra the P.E.S.T. Winsim software (http://epr.niehs.nih.gov/; versions 0.964, v0.98, or 1.02002) was used.

*Pulse gradient spin echo‐NMR measurements* were performed on an Avance III HD spectrometer (Bruker) operating at 700.37 MHz for 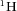, using a TCI prodigy cryoprobe head equipped with a *z*‐gradient unit. The gradient was calibrated using “doped water” (1% $H_{2}O$ in $D_{2}O$ with traces of ${\mathrm{CuSO}}_{4}$) assuming a diffusion coefficient of $1.91\times 10^{-5}$ ${\mathrm{cm}}^{2}$ $s^{-1}$ for HDO. The diffusion measurements used a modified bipolar gradient pulse pair‐stimulated echo sequence incorporating a longitudinal eddy current delay (BPP‐LED). The gradient pulse length (δ) and the diffusion time (Δ) were kept at fixed values while gradually increasing the gradient strength. Typical values for δ and Δ were 2 and 50 ms, respectively. A longitudinal eddy current delay ($T_{e}$) of 5 ms was used. Sine‐shaped gradient pulses were linearly varied between 1 and 52 G ${\mathrm{cm}}^{-1}$ (2% to 98%) in 32 steps and at each step 32 scans were acquired. Five measurements per sample were performed at a constant sample temperature of 290 ± 0.1 K (Bruker Variable Temperature Unit BCU II).

The data were analyzed with the $T_{1}$/$T_{2}$ relaxation module of Topspin 3.5. The signal areas were plotted against the gradient strength and the best fit was calculated using the Stejskal–Tanner equation

(2) $$I_{g}=I_{0}\times \exp\left[-4\pi^{2}\gamma^{2}\delta^{2}G^{2}\left(\Delta -\delta /3\right)\right]D$$

(with D being the diffusion coefficient in ${\mathrm{cm}}^{2}$ $s^{-1}$, γ the gyromagnetic ratio in Hz/G, G the gradient strength in G ${\mathrm{cm}}^{-1}$, δ the gradient length in ms, Δ the interval between gradient pulses (diffusion time) in ms, $I_{g}$ the signal area, and $I_{0}$ the signal intensity at G−0%). Mean values for each sample are reported.

## Conflicts of Interest

The authors declare no conflicts of interest.

## Supporting information

The authors have cited additional references within the Supporting Information [89, 90, 91, 92, 93, 94, 95, 96, 97, 98, 99, 100, 101, 102, 103]. The Supporting Information includes the following items: Selected additional cyclic voltammetric data (example for the effect of background correction; peak potential differences as a function of concentration and scan rate for selected experiments for **2** and **4** in 0.1 M ${\mathrm{NBu}}_{4}$ ${\mathrm{PF}}_{6}$/${\mathrm{CH}}_{2}$ ${\mathrm{Cl}}_{2}$; cyclic voltammogram of **2** in 0.1 M ${\mathrm{NBu}}_{4}$ ${\mathrm{PF}}_{6}$/${\mathrm{CH}}_{2}$ ${\mathrm{Cl}}_{2}$ in the reductive potential region at small scan rate; discussion of second oxidation processes of **2** and **4** including cyclic voltammograms and ratios of oxidation peak currents as a function of scan rate); computational methods; results of density functional calculations. Additional supporting data are deposited in an archive of the Cartesian coordinates [104].

## Endnotes

2 A definition of the terms “qualitative” and “quantitative” as used in the context of cyclic voltammetric analysis in molecular electrochemistry is presented in ref. [88].
