# Supplementary material for: 5,12‐TIPS‐Substitution of Tetracene: Effects on Redox Potentials and Cyclic Voltammetric Behavior in CH2Cl2 and THF
Source: Chemistry. 2026 May 9;32(18):e03591. doi: 10.1002/chem.202503591 (PMC13174928; doi:10.1002/chem.202503591)
Supplement: Supplementary file 1 — The authors have cited additional references within the Supporting Information [89, 90, 91, 92, 93, 94, 95, 96, 97, 98, 99, 100, 101, 102, 103]. The Supporting Information includes the following items: Selected additional cyclic voltammetric data (example for the effect of background correction; peak potential differences as a function of concentration and scan rate for selected experiments for 2 and 4 in 0.1 M NBu4 PF6/CH2 Cl2; cyclic voltammogram of 2 in 0.1 M NBu4 PF6/CH2 Cl2 in the reductive potential region at small scan rate; discussion of second oxidation processes of 2 and 4 including cyclic voltammograms and ratios of oxidation peak currents as a function of scan rate); computational methods; results of density functional calculations. Additional supporting data are deposited in an archive of the Cartesian coordinates [104]. [file CHEM-32-e03591-s001.pdf]

Supporting Information for 5,12-TIPS-Substitution of Tetracene: Effects on Redox Potentials and Cyclic Voltammetric Behavior

Holger F. Bettinger,[a] Simon Schundelmeier,[a] Bernd Speiser[a]*

April 22, 2026

The numbering of compounds and the use of symbols in these Supporting Informations is identical to that in the main manuscript.

*corresponding author

[a]Prof. Dr. Holger F. Bettinger, Dr. S. Schundelmeier, Prof. Dr. B. Speiser, Universität Tübingen, Institut für Organische Chemie, Auf der Morgenstelle 18, D–72076 Tübingen, Germany; bernd.speiser@uni-tuebingen.de

Selected Additional Cyclic Voltammetry Data

Example for Background Correction

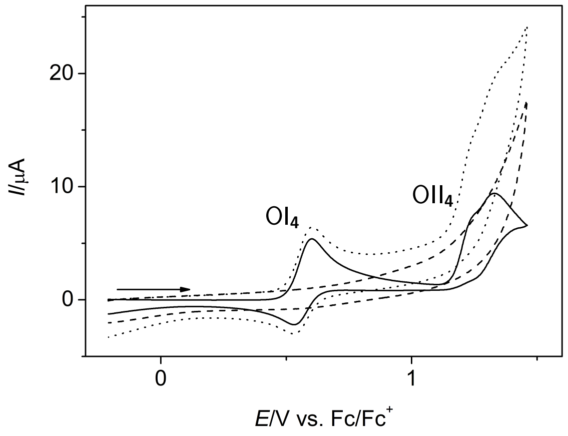

Figure S1: Effect of background correction for cyclic voltammetry of TIPS-tetracene **4** in 0.1 M NBu_4PF_6/CH_2Cl_2 in the potential range of oxidation, scan rate v = 0.2 V s^{-1}, c = 0.25 mM; dotted line: original, uncorrected voltammogram, broken line: background voltammogram, full line: corrected voltammogram after subtraction of background.

Peak Potential Differences

Table S1: Peak potential differences $\Delta E_{\mathrm{p}}(\mathrm{OI}_{\mathbf{4}})$ in 0.1 M NBu_4PF_6/CH_2Cl_2 from an exemplary experiment.

	$c(\mathbf{4})$/mM			
v/V s^{-1}	0.119	0.154	0.189	0.210
0.02	0.066	0.067	0.067	0.067
0.05	0.065	0.065	0.065	0.065
0.1	0.065	0.066	0.065	0.067
0.2	0.065	0.066	0.067	0.068
0.5	0.068	0.070	0.070	0.072
1.0	0.069	0.073	0.071	0.073
2.0	0.076	0.076	0.078	0.079
5.0	0.082	0.086	0.087	0.089
10	0.095	0.097	0.101	0.103
21	0.109	0.117	0.121	0.121
35	0.131	0.134	0.137	0.140

Table S2: Peak potential differences $\Delta E(\mathrm{OI}_\mathbf{2})$ during oxidation of **2** in 0.1 M NBu_4PF_6/CH_2Cl_2 from an exemplary experiment.

	$c(\mathbf{2})$/mM			
v/V s^{-1}	0.159	0.185	0.206	0.222
0.02	0.070	0.071	0.071	0.071
0.05	0.067	0.066	0.066	0.066
0.1	0.066	0.066	0.066	0.066
0.2	0.066	0.066	0.066	0.066
0.5	0.068	0.068	0.068	0.068
1.0	0.068	0.068	0.070	0.070
2.0	0.071	0.071	0.072	0.074
5.0	0.078	0.079	0.082	0.083
10	0.086	0.090	0.095	0.095
21	0.103	0.107	0.113	0.113
34	0.122	0.128	0.134	0.134

Reduction of 2 at Slow Scan Rates

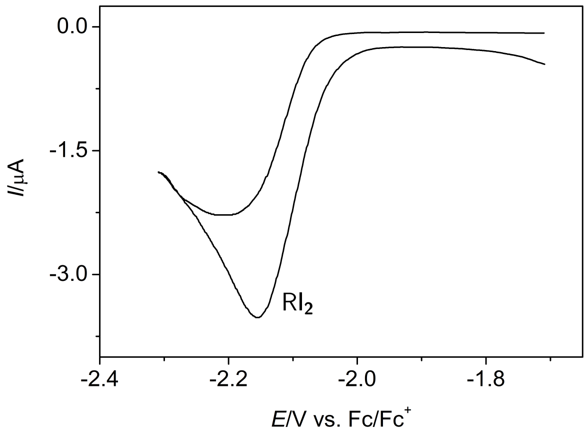

Figure S2: Cyclic voltammetry of tetracene **2** in 0.1 M NBu_4PF_6/CH_2Cl_2 in the potential range of reduction, slow scan rate $v = 0.02$ V s^{-1}, $c = 0.19$ mM.

Second Oxidation Processes of Tetracene and TIPS-Tetracene

The second oxidation of tetracene and TIPS-tetracene can be observed in CH_2Cl_2 electrolytes at potentials above 1 V (signals OII in Figures S3 and S4).

For the substituted compound **4** and at slow scan rates, signal $\mathrm{OII}_{\mathbf{4}}^{\mathrm{ox}}$ appears to be composed of two peaks without indication of a reverse peak (Figure S3a for the example of $v = 0.05$ V s^{-1}), while at fast scan rates the maxima merge and a weak signal is observed on the reverse scan (Figure S3b for the example of $v = 5$ V s^{-1}). Scan rate normalized cyclic voltammograms (current divided by $v^{1/2}$) for various v at a particular $c(\mathbf{4})$ are compared in Figure S3c and show the changes of the peak shape for $\mathrm{OII}_{\mathbf{4}}^{\mathrm{ox}}$ with the time scale of the experiment. In this type of presentation, the curves in the region of peak $\mathrm{OI}_4^{\mathrm{ox}}$ at all v coincide as expected for a nearly reversible process, where the currents scale with $v^{1/2}$. The ratio of the peak currents of $\mathrm{OII}_4^{\mathrm{ox}}$ and $\mathrm{OI}_4^{\mathrm{ox}}$ is approximately 2 at slower scan rates and decreases with increasing v to 1.3 (Table S3). This indicates a kinetic component in process OII_4, in particular this second oxidation process must be some type of an ECE sequence, involving two electrons at small v (where the C step proceeds to almost completion close to the electrode within the experimental time scale) and one electron at large v (where the chemical reaction is so slow that the oxidation of its product contributes only a minor current component). The formal potentials of the two E steps must be rather close. In addition, there must be an irreversible reaction component in the overall mechanism, to account for the small extent of the reverse peak even at 5 V s^{-1}. This behavior of $\mathrm{OII}_{\mathbf{4}}$ in cyclic voltammetry is similar to the mechanism governing the second oxidation of TIPS-pentacene **5** in 0.1 M NBu_4PF_6/CH_2Cl_2, which was supported by extensive reaction-diffusion simulations.[1] However, for **5**, the peak current ratio $I_\mathrm{p}^{\mathrm{ox}}(\mathrm{OII}_{\mathbf{5}})/I_\mathrm{p}^{\mathrm{ox}}(\mathrm{OI}_{\mathbf{5}})$ reaches a limiting value of unity at large v. Then, the second oxidation of $\mathbf{5}^{\bullet+}$ to $\mathbf{5}^{2+}$ corresponds to a one-electron step. Also, the reverse signal for **5** indicates the stability of $\mathbf{5}^{2+}$ at fast time scales (large v). It is visible even at small v (0.02 V s^{-1}). In contrast, for **4**, process $\mathrm{OII}_{\mathbf{4}}$ occurs at potentials which are several 100 mV more positive than in the case of $\mathrm{OII}_{\mathbf{5}}$, and is located close to the positive limit of the potential window. This precludes a further quantitative analysis of the peak current data. The voltammograms show, however, that for TIPS-tetracene at least the oxidation state of a dication $\mathbf{4}^{2+}$ can be reached. The structure and identity of any follow-up products can not be deduced from the electroanalytical data.

The second oxidation peak of the unsubstituted **2** has a totally (chemically) irreversible shape without a reverse peak for all scan rates used in our experiments (see Figures S4a and b for 0.05 and 1.0 V s^{-1}). The irreversible reaction component must be faster than for **4**, where we did just start to observe a reverse signal at high scan rates. A splitting of peak $\mathrm{OII}_{\mathbf{2}}^{\mathrm{ox}}$ is not found, but again the current ratio of the two oxidation peaks, here $I_\mathrm{p}^{\mathrm{ox}}(\mathrm{OII}_{\mathbf{2}})/I_\mathrm{p}^{\mathrm{ox}}(\mathrm{OI}_{\mathbf{2}})$, depends on the time scale of the experiment and even reaches unity for fast scan rates (Table S4). At small v, it increases again to a value of 2, as a result of the ECE type reaction. The comparison of scan rate normalized cyclic voltammograms (Figure S4c) shows the typical features already seen for **4**. However, in addition, the peak potential $E_\mathrm{p}(\mathrm{OII}_{\mathbf{2}}^{\mathrm{ox}})$ shifts to more positive potentials with increasing v. This is typical for follow-up chemical reactions coupled to an electron transfer.[2] The absence of a split double oxidation peak at small v indicates that the potentials of the two steps are closer as compared to the case of **4**. We note that an unsubstituted tetracene dication $\mathbf{2}^{2+}$ has recently been obtained and even crystallized after chemical oxidation by a perfluoronaphthalene radical cation salt as a powerful oxidant.[3]

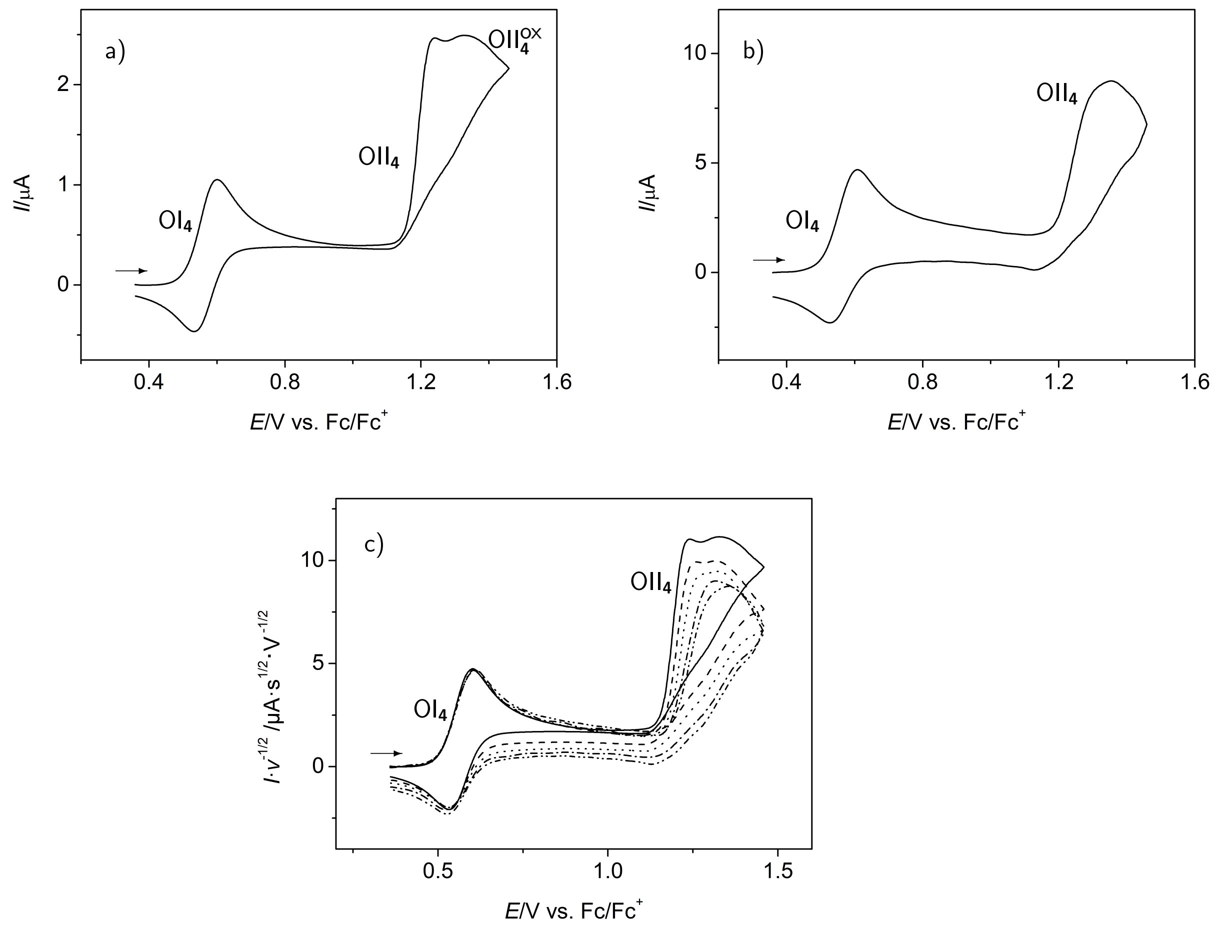

Figure S3: Cyclic voltammetry of **4** in the potential range of first and second oxidation processes $OI_{\mathbf{4}}$ und $OII_{\mathbf{4}}$ in 0.1 M NBu_4PF_6/CH_2Cl_2; arrows indicate the beginning of the potential scan; a) cyclic voltammogram at $v = 0.05$ V s^{-1}, $c = 0.09$ mM; b) cyclic voltammogram at $v = 5.0$ V s^{-1}; $c = 0.09$ mM; c) scan rate normalized cyclic voltammograms at $v = 0.05$ V s^{-1} (—), 0.1 V s^{-1} (- - -), 0.2 V s^{-1} (· · ·), 0.5 V s^{-1} (— · —·), 1.0 V s^{-1} (— · · — ··), $c = 0.09$ mM.)

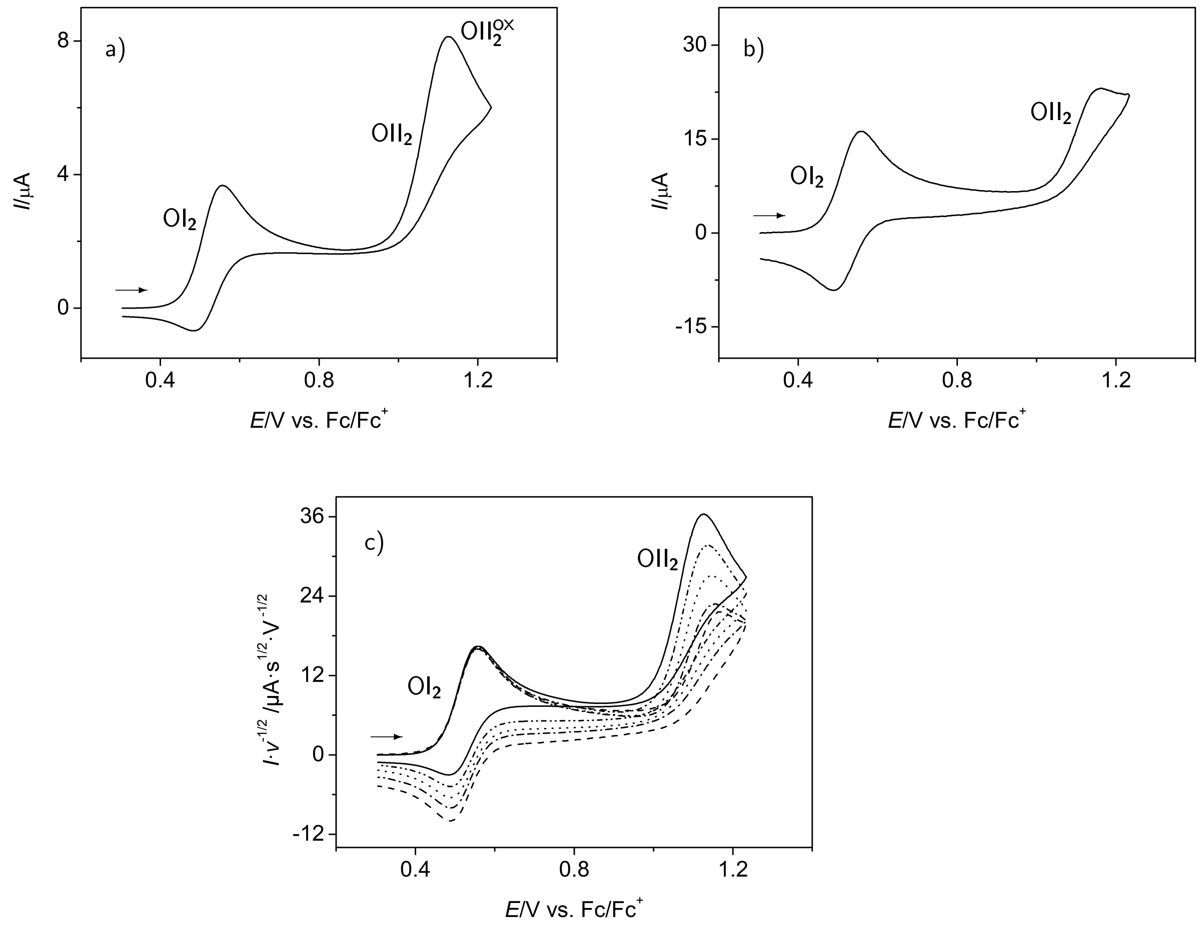

Figure S4: Cyclic voltammetry of **2** in the potential range of first and second oxidation processes OI_2 and OII_2 in 0.2 M TBAHFP/CH_2Cl_2; arrows indicate the beginning of the potential scan; a) cyclic voltammogram at $v = 0.05$ V s^{-1}, $c = 0.22$ mM; b) cyclic voltammogram at $v = 1.0$ V s^{-1}, $c = 0.22$ mM; c) scan rate normalized cyclic voltammograms at $v = 0.05$ V s^{-1} (—), 0.1 V s^{-1}(− · · − · · − · ·), 0.2 V s^{-1} (· · ·), 0.5 V s^{-1} (− · −·), 2.0 V s^{-1} (− − −), $c = 0.22$ mM.

Table S3: Peak current ratio $I_p^{ox}(OII_{\mathbf{4}})/I_p^{ox}(OI_{\mathbf{4}})$ in voltammograms of **4** in 0.1 M NBu_4PF_6/CH_2Cl_2 at various scan rates; $I_p^{ox}(OII_{\mathbf{4}})$ was determined relativ to the extrapolated current decreasing from the oxidation peak of process $OI_{\mathbf{4}}$; mean values from two experiments with a total of 9 concentrations ($0.031 \leq c/\text{mM} \leq 0.15$).

v	$I_p^{ox}(OII_{\mathbf{4}})/I_p^{ox}(OI_{\mathbf{4}})$
0.02	2.1 ± 0.1
0.05	1.9 ± 0.1
0.1	1.8 ± 0.1
0.2	1.6 ± 0.1
0.5	1.5 ± 0.1
1.0	1.4 ± 0.1
2.0	1.4 ± 0.1
5.0	1.3 ± 0.1

Table S4: Peak current ratio $I_p^{ox}(OII_{\mathbf{2}})/I_p^{ox}(OI_{\mathbf{2}})$ at various scan rates; for the calculation of the mean values for the 0.1 M electrolyte two experiments with a total of 8 concentrations ($0.17 \leq c/\text{mM} \leq 0.32$) were used, while in the case of the 0.2 M electrolyte two experiments with a total of 10 concentrations ($0.04 \leq c/\text{mM} \leq 0.26$) were analyzed.

v/V s^{-1}	0.1 M NBu_4PF_6/ CH_2Cl_2	0.2 M NBu_4PF_6/ CH_2Cl_2
0.05	1.9 ± 0.03	1.9 ± 0.06
0.1	1.7 ± 0.05	1.7 ± 0.05
0.2	1.6 ± 0.09	1.5 ± 0.06
0.5	1.3 ± 0.05	1.3 ± 0.09
1	1.1 ± 0.07	1.1 ± 0.12
1.993	1.0 ± 0.09	1.0 ± 0.10
5	–	1.0 ± 0.12

Computation of Ionization Potentials of Tetracene 2 and TIPS-Tetracene 4

Computational Methods

Conformer sampling and sorting for tetracene and TIPS-tetracene was done with the CREST program version 2.12 using the default iMTD-GC workflow and the semi-empirical extended tight-binding method GFN2-xTB.[4–6] The effect of the solvents CH_2Cl_2 and THF was taken into account using the analytical linearized Poisson-Boltzmann model (ALPB) as a robust and efficient implicit solvation model.[7]

All conformers within an energy window of 6 kcal/mol at a temperature of $T = 298.15$ K were subject to subsequent ranking and refinement using the CENSO program, version 2.1.4.[8] The temperature was set to $T = 298.15$ K, and the impact of solvent was modelled using ALPB. Note that the impact of the electrolyte was ignored.

The CENSO protocol included *prescreening* (done at the PBE-D4/def2-SV(P) level;[9–12] energy threshold for discarding conformers was 4.0 kcal/mol), *screening* (done at the r^{2}SCAN-3c level[13]; energy threshold for discarding conformers was 3.5 kcal/mol), *geometry optimization* (done at the r^{2}SCAN-3c level; energy threshold for discarding conformers was 3.0 kcal/mol), and *refinement* (done at the ωB97X-V/def2-TZVP[12,14] level with a Boltzmann sum threshold of 95%). The Boltzmann average at $T = 298.15$ K gives the final ensemble energy avG_{tot} given in Table S5. In addition, the individual electronic energy E, free energy of solvation δG_{solv}, and free energy of thermostatistical contributions G_{mRRHO} of the most stable conformer along with its weight to the averaged ensemble is given in Table S5. According to equation 1, the sum of these terms gives the free energy of this conformer:

$$G = E + \delta G_{\text{solv}} + G_{\text{mRRHO}} \quad (1)$$

The quantum chemical computations utilized the XTB 6.4.1[15] and Orca[16,17] programs.

Numerical Computational Results

Numerical results of the computations are given in Table S5 on the following page. In addition, an archive of the Cartesian coordinates is available at RADAR4Chem under the DOI 10.22000/pq3hnekvzqeert17.

Table S5: Total DFT energies E(DFT) of tetracene (**2**) and TIPS-tetracene (**4**) species (in Hartree), free energy of solvation δG_{solv} (in Hartree) and free energy contribution in the rigid rotator, harmonic oscillator approximation G_{mRRHO} (in Hartree), total free energy G_{tot} (in Hartree) and energy differences Δ between ion and neutral (in eV) in the gas phase, CH_2Cl_2, and THF. For **4** species the Boltzmann averaged free energy (avG_{tot}) is given (in Hartree), while E(DFT), δG_{solv}, G_{mRRHO}, and G_{tot} are given for the lowest energy conformer whose number and weight in the ensemble is also given ($T = 298.15$ K). Differences in G_{tot} or avG_{tot} correspond to electron affinities for anions and ionization energy for cations. $\Delta\delta G_{\mathrm{solv}}$ (in eV) is the difference between δG_{solv} of **4** and **2**.

	Neutral/E_h	Anion/E_h	Δ/eV	Cation/E_h	Δ/eV	Neutral/E_h	Anion/E_h	Δ/eV	Cation/E_h	Δ/eV	Neutral/E_h	Anion/E_h	Δ/eV	Cation/E_h	Δ/eV
	Gas phase					CH_2Cl_2					THF				
**2**															
E(DFT)	−693.18564	−693.22090	−0.96	−692.93997	6.69	−693.18555	−693.22085	−0.96	−692.93996	6.68	−693.18556	−693.22086	−0.96	−692.93997	6.68
δG_{solv}						−0.02553	−0.08084	−1.51	−0.08154	−1.52	−0.02106	−0.07485	−1.46	−0.07566	−1.49
G_{mRRHO}	0.19507	0.19209	-0.10	0.19378	0.0	0.19479	0.19185	−0.08	0.19356	−0.03	0.19462	0.19178	−0.08	0.19351	−0.03
G_{tot}	−692.99057	−693.02881	**−1.04**	−692.74619	**6.65**	−693.01628	−693.10985	**−2.55**	−692.82795	**5.12**	−693.01200	−693.10393	**−2.50**	−692.82212	**5.17**
**4**															
E(DFT)	−2134.57044	−2134.63561	−1.77	−2134.33508	6.40	−2134.57054	−2134.63364	−1.72	−2134.33664	6.36	−2134.57157	−2134.63440	−1.71	−2134.33599	6.41
δG_{solv}						−0.04014	−0.08858	−1.32	−0.08304	−1.17	−0.03129	−0.07804	−1.27	−0.07337	−1.15
G_{mRRHO}	0.70216	0.70128	−0.02	0.69852	−0.10	0.70072	0.69887	−0.05	0.69900	−0.05	0.70185	0.69987	−0.05	0.69890	−0.08
G_{tot}	−2133.86828	−2133.93433	−1.80	−2133.63656	6.31	−2133.90996	−2134.02335	−3.09	−2133.72067	5.15	−2133.90101	−2134.01256	−3.04	−2133.71046	5.19
avG_{tot}	−2133.86721	−2133.93289	**−1.79**	−2133.63526	**6.31**	−2133.90849	−2134.02160	**−3.08**	−2133.71973	**5.14**	−2133.89992	−2134.01099	**−3.02**	−2133.70947	**5.18**
lowest conf.	conf202	conf2		conf204		conf84	conf104		conf10		conf2	conf25		conf25	
Weight	11.78	30.43		19.65		17.25	28.44		16.96		16.13	29.95		14.87	
$\Delta\delta G_{\mathrm{solv}}$						−0.40	−0.21		−0.04		−0.28	−0.09		0.06	

References

[1] S. Schundelmeier, B. Speiser, H.F. Bettinger, R. Einholz, *ChemPhysChem* **2017**, *18*, 2266.

[2] R.S. Nicholson, I. Shain, *Anal. Chem.* **1964**, *36*, 706.

[3] M. Sellin, J. Willrett, D. Röhner, T. Heizmann, J. Fischer, M. Seiler, C. Holzmann, T.A. Engesser, V. Radtke, I. Krossing, *Angew. Chem. Int. Ed.* **2024**, *63*, e202406742.

[4] C. Bannwarth, S. Ehlert, S. Grimme, *J. Chem. Theory Comput.* **2019**, *15*, 1652.

[5] S. Grimme, *J. Chem. Theory Comput.* **2019**, *15*, 2847.

[6] P. Pracht, F. Bohle, S. Grimme, *Phys. Chem. Chem. Phys.* **2020**, *22*, 7169.

[7] S. Ehlert, M. Stahn, S. Spicher, S. Grimme, *J. Chem. Theory Comput.* **2021**, *17*, 4250.

[8] S. Grimme, F. Bohle, A. Hansen, P. Pracht, S. Spicher, M. Stahn, *J. Phys. Chem. A* **2021**, *125*, 4039.

[9] J.P. Perdew, K. Burke, M. Ernzerhof, *Phys. Rev. Lett.* **1996**, *77*, 3865.

[10] J.P. Perdew, K. Burke, M. Ernzerhof, *Phys. Rev. Lett.* **1997**, *78*, 1396.

[11] E. Caldeweyher, C. Bannwarth, S. Grimme, *J. Chem. Phys.* **2017**, *147*, 034112.

[12] F. Weigend, R. Ahlrichs, *Phys. Chem. Chem. Phys.* **2005**, *7*, 3297.

[13] S. Grimme, A. Hansen, S. Ehlert, J.-M. Mewes, *J. Chem. Phys.* **2021**, *154*, 064103.

[14] N. Mardirossian, M. Head-Gordon, *Phys. Chem. Chem. Phys.* **2014**, *16*, 9904.

[15] C. Bannwarth, E. Caldeweyher, S. Ehlert, A. Hansen, P. Pracht, J. Seibert, S. Spicher, S. Grimme, *WIREs Computational Molecular Science* **2021**, *11*, e1493.

[16] F. Neese, *WIREs Computational Molecular Science* **2012**, *2*, 73.

[17] F. Neese, *WIREs Computational Molecular Science* **2022**, *12*, e1606.
